# PVT1‐104aa derived from the 8q24 gene desert promotes colorectal cancer tumorigenesis

**DOI:** 10.1002/ctm2.70654

**Published:** 2026-04-08

**Authors:** Maoguang Ma, Mingdian Wang, Yufei Yang, Dakui Luo, Weixing Dai, Yiwei Li, Sanjun Cai, Shaobo Mo, Qingguo Li, Xinxiang Li

**Affiliations:** ^1^ Department of Colorectal Surgery Fudan University Shanghai Cancer Center Shanghai China; ^2^ Department of Oncology Shanghai Medical College, Fudan University Shanghai China; ^3^ Department of Pathology Zhongshan Hospital, Fudan University Shanghai China

**Keywords:** circRNA, c‐Myc ubiquitination, colorectal cancer, combination immunotherapy, PVT1‐104aa oncopeptide

## Abstract

**Background:**

Current therapeutic outcomes for advanced colorectal cancer (CRC) remain suboptimal, and chemotherapy‐based regimens continue to be the mainstay of treatment. Circular RNAs (circRNAs) can serve as templates for translating short peptides or proteins, and the resulting products actively regulate malignant tumour progression, making them attractive therapeutic targets.

**Methods:**

We identified the novel protein PVT1‐104aa translated from circPVT1, which is generated by back‐splicing of the non‐coding *PVT*1 gene. Its expression and clinical significance were examined in CRC clinical specimens and cell lines. Proliferation, metastasis, and tumour growth were assessed by CCK‐8, colony formation, transwell, wound healing, and xenograft syngeneic tumour models. Mechanistic studies were performed by immunoprecipitation, ubiquitination assays, and protein half‐life analysis. The relationship between PVT1‐104aa, c‐Myc, and PD‐L1 was evaluated by promoter reporter assays, ChIP‐qPCR, and immunohistochemistry. The therapeutic efficacy of combining PVT1‐104aa inhibition with anti‐PD‐L1 therapy was tested in vivo.

**Results:**

PVT1‐104aa was significantly overexpressed in CRC and correlated with poor patient prognosis. Functionally, it drove tumour progression by promoting proliferation and metastasis. Mechanistically, PVT1‐104aa enhanced c‐Myc phosphorylation at Ser62, disrupted the c‐Myc‐FBW7 interaction, and thereby inhibited ubiquitin‐mediated degradation of c‐Myc, as shown by accelerated c‐Myc turnover upon PVT1‐104aa knockdown. In addition, PVT1‐104aa regulated PD‐L1 expression through c‐Myc. Combining anti‐PVT1‐104aa with anti‐PD‐L1 therapy suppressed CRC growth and increased CD4^+^ and CD8^+^ T cell infiltration in xenograft syngeneic tumour models and CRC tissues.

**Conclusions:**

Our results uncover a pathogenic role of the PVT1‐originated molecular species PVT1‐104aa and suggest that targeting this pathway represents a promising therapeutic strategy for CRC treatment.

**Key points:**

- Circ‐PVT1 is upregulated in CRC patients and encoded a novel protein: PVT1‐104aa.
- PVT1‐104aa promotes CRC progression and predicts worse prognosis.
- PVT1‐104aa enhance Myc expression through inhibition of Myc ubiquitination.
- PVT1‐104aa regulate PD‐L1 expression of CRC cells and modulate T cells infiltration in vivo.

## Graphical abstract

The schematic illustrates the dual oncogenic mechanism of circPVT1‐encoded PVT1‐104aa in CRC. First, MYC stabilization: (1) Direct binding to MYC; (2) phosphorylation of MYC at Ser62; (3) protection from GSK3β/FBW7‐mediated ubiquitination. Second, immunomodulation: (1) MYC‐dependent PD‐L1 upregulation; (2) enhanced therapeutic efficacy when combining PVT1‐104aa inhibition with anti‐PD‐L1 treatment in xenograft models.

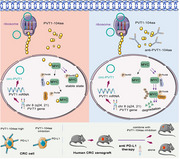

## 1 INTRODUCTION

Colorectal cancer (CRC) exhibits a high incidence rate, and with advancements in diagnostic and therapeutic approaches, treatment outcomes have significantly improved. However, patients with metastatic colorectal cancer still owned poor prognosis, with fewer than 20% survived beyond 5 years.^1^ This suggests that developing novel drug targets is crucial for improving therapeutic efficacy in advanced CRC.

*MYC* (also termed as c‐Myc) is a pivotal oncogene that drives tumorigenesis and therapeutic resistance in CRC.^2^, ^3^ The *MYC* gene resides within the 8q24.21 chromosomal region, a characterized ‘gene desert’ dominated by non‐coding elements including *PVT1*.^4^, ^5^ Copy number gains (CNGs) of the 8q24 chromosomal region are a frequent occurrence across multiple cancer types and are strongly associated with tumour prognosis.^6^, ^7^ Moreover, co‐amplification and co‐operation of *MYC* and *PVT1* drives malignant transformation of pleural mesothelioma and CRC.^8^, ^9^

Circular RNAs (circRNAs) play a significant role in cancer progression.^10^, ^11^ Their primary biological function involves serving as microRNA sponges to regulate gene expression.^12^ In addition, circRNAs can function as templates for protein translation, and these proteins may exert either oncogenic or tumour‐suppressive biological functions in cancer.^13^, ^14^ CircPVT1 is generated through the back‐splicing of the second exon within the *PVT1* gene locus. As reported by Panda et al.,^15^ circPVT1 functions as a senescence‐associated circular RNA that binds with the microRNA let‐7, thereby derepressing let‐7 target genes (including IGF2BP1, KRAS and HMGA2) to modulate senescence in human fibroblasts. Moreover, circPVT1 promotes the progression of malignant tumours. Yi et al.^16^ reported that circPVT1 promotes ER‐positive breast carcinogenesis and endocrine therapy resistance through dual mechanisms: it functions as a miRNA sponge to sequester miR‐181a‐2‐3p and upregulate ESR1 expression, while also serving as a protein scaffold that binds MAVS and disrupts RIGI‐MAVS complex formation. According to findings by Wang and colleagues, circPVT1 facilitates gallbladder carcinoma (GBC) advancement through sequestering miR‐339‐3p, consequently enhancing Myeloid cell leukaemia‐1 (MCL‐1) protein levels.^17^ However, it remains unknown whether circPVT1 has protein‐coding potential and how its putative translational products might influence tumour biology.

Here, we give evidence that PVT1‐104aa, encoded by circPVT1 (a circRNA derived from the non‐coding gene *PVT1*), is overexpressed in CRC tissues.PVT1‐104aa upregulation is linked to deteriorated prognosis among individuals with CRC. The protein product PVT1‐104aa—rather than its circPVT1 precursor—drives proliferative and metastatic capacities in CRC. Mechanistically, PVT1‐104aa stabilizes c‐Myc by inhibiting its ubiquitin‐mediated degradation and subsequently modulates PD‐L1 expression through c‐Myc‐dependent regulation. This finding is consistent with previous literature reports demonstrating ‘a close functional relationship between the *MYC* and *PVT1* in promoting tumorigenesis.’ In vivo studies demonstrate that combinatorial therapy targeting PVT1‐104aa with anti‐PD‐L1 antibodies elicits potent antitumour efficacy. These findings suggest that PVT1‐104aa may represent both a novel therapeutic target and an immune regulatory molecule in CRC, establishing a theoretical framework for developing more effective treatments for advanced‐stage disease.

## 2 RESULTS

### 2.1 Identification of circPVT1 as a potential tumour promoter in CRC

**FIGURE 1:**
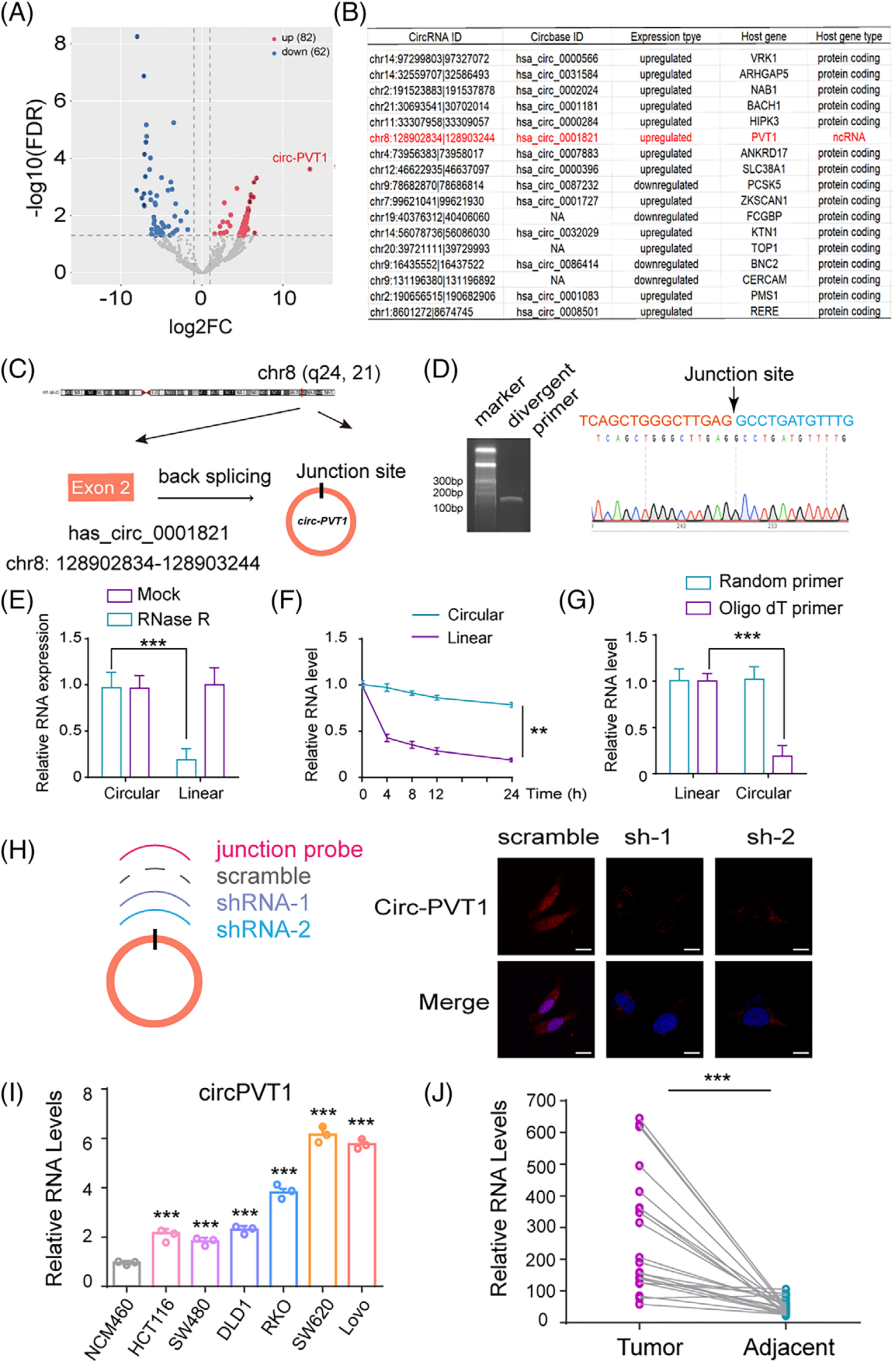
Identification of circPVT1 as a potential tumour promoter in CRC. (A) Volcano plot displays circRNA expression changes in 4 paired CRC/normal samples from RNA sequencing. (B) The seventeen significantly up‐ and down‐expressed circRNAs were predicted to encode peptides using circRNADb's multi‐parameter algorithm. (C) Genomic annotation of the PVT1 locus showing putative mRNA splicing variants and circPVT1 formation. (D) The back‐splicing site of in circPVT1 was confirmed by Sanger sequencing from Lovo cells using divergent primers. (E) RNase R resistance assay comparing circPVT1 and linear PVT1 stability. (F) Time‐course analysis of circPVT1 and linear PVT1 RNA stability. (G) qPCR detection of circPVT1 and linear PVT1 in SW480 cells using oligo‐dT or random primers for reverse transcription. (H) Left, schematic of circPVT1 junction‐specific probe and shRNAs (sh1, sh2); Right, FISH detection of circPVT1 in modified Lovo cells (scale bar: 10 µm). (I) Relative circPVT1 expression levels were detected in NCM460 cells and six CRC cell lines as indicated. (J) circPVT1 expression in 24 paired CRC tumours versus normal tissues. Ns, no statistically significant, ***p < .001.

To identify differentially expressed circRNAs in CRC, RNA sequencing was conducted on four clinical CRC specimens along with matched adjacent normal tissues (Figure 1A). Comprehensive transcriptomic profiling revealed 5970 circular RNAs, including 144 differentially expressed candidates between colorectal carcinoma and matched normal mucosa (False Discovery Rate FDR<.05, |log2FC|≥1; 62 down and 82 upregulated in tumour; Table S1). Functional annotation through the KEGG demonstrated that these targeted genes were enriched in Wnt, PI3K‐AKT, Focal adhesion and JAK‐STAT pathways, findings concordant with the predominant signalling activation patterns in CRC (Figure S1A). Then, we matched the significantly differentially expressed circRNAs with circRNADb to screen potential translatable circRNAs. 17 circRNAs were identified in total; however, circPVT1 was the only circRNA with protein‐coding potential and an untranslatable host gene (Figure 1B). Further investigation was subsequently directed towards circPVT1. CircPVT1 was identified as deriving from circularization of exon 2 in the *PVT1* gene based on genomic locus examination (Figure 1C). Experimental validation employing divergent primer‐based amplification and subsequent Sanger sequencing confirmed the circular junction of circPVT1, showing complete consistency with the reference sequence in circBase (ID: has_circ_0001821, Figure 1D). Enhanced molecular stability was observed for circPVT1, demonstrated by its RNase R resistance and prolonged half‐life compared with *PVT1* mRNA (Figure 1E,F). Divergent detection patterns were observed in SW480 cells: circPVT1 amplification occurred exclusively with random primers, demonstrating its non‐polyadenylated nature, while linear PVT1 was detectable with both primer types (Figure 1G). Specific targeting of the circPVT1 backsplice junction was achieved using 2 shRNAs complementary to the unique circularization site (Figure 1H, left). Knockdown efficiency was confirmed in LoVo cells using a junction‐specific probe. Furthermore, fluorescence in situ hybridization (FISH) and cell fraction qPCR revealed that circPVT1 exhibits dual localization, distributing in both the cytoplasm and nucleus (Figure 1H, right; Figure S1B). Furthermore, we confirmed that circPVT1 expression is dependent on PVT1 mRNA levels (Figure S1C,D). Expression profiling revealed marked elevation of circPVT1 levels in multiple CRC cell lines (e.g., Lovo, SW620) compared with normal human intestinal epithelial cells (Figure 1I). Analysis of a clinical cohort comprising 24 randomly selected paired CRC specimens revealed elevated circPVT1 expression in cancerous tissues relative to their paired adjacent non‐cancerous counterparts (Figure 1J). These results collectively suggest that circPVT1 is upregulated in CRC and functions as an oncogenic circRNA.

### 2.2 CircPVT1 encodes a 104‐amino‐acid protein

**FIGURE 2:**
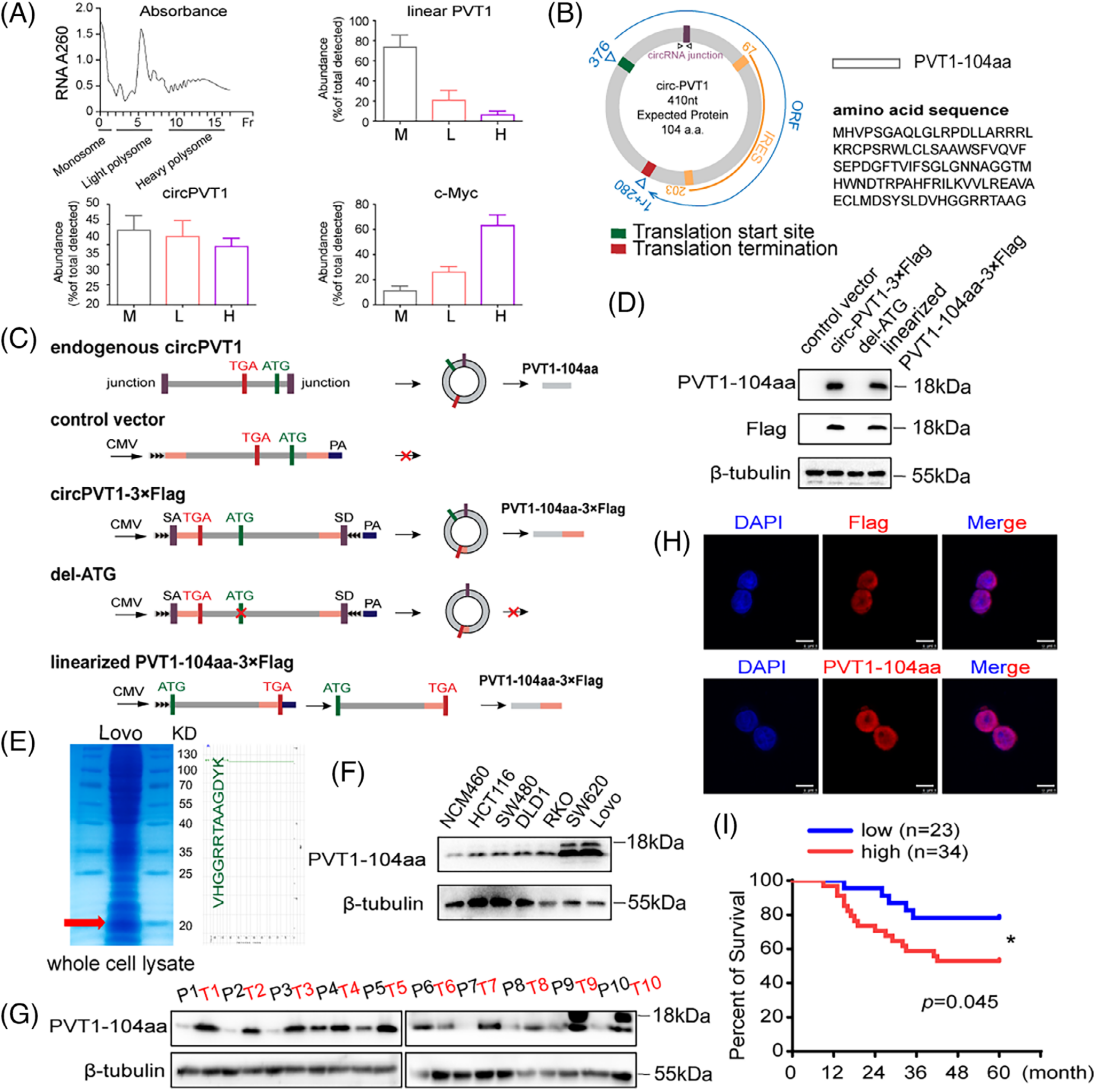
CircPVT1 encodes a novel 104‐amino acid protein (PVT1‐104aa). A Polysome profiling of 293T cells transfected with the circPVT1 plasmid, mRNA of c‐Myc and PVT1 as positive and negative controls, respectively. CircPVT1 distribution across fractions was analyzed by qPCR. (B) Schematic of PVT1‐104aa peptide features: ORF, IRES element, start/stop codons and complete amino acid sequence. (C) Construct designs: endogenous circPVT1, control vector, circPVT1‐3×Flag, ATG‐deletion mutant (del‐ATG) and linearized PVT1‐104aa‐3×Flag. (D) Western blot analysis of cells expressing the indicated plasmids with antibodies as indicated. (E) Mass spectrometry identification of endogenous PVT1‐104aa peptide sequences in Lovo cells. (F) PVT1‐104aa expression levels in normal colon epithelial cells (NCM460) versus six CRC cell lines. (G) The expression of PVT1‐104aa in 10 clinical paired CRC samples by immunoblotting. (H) Immunofluorescence detection of circPVT1‐3×Flag‐transfected cells with antibodies as indicated (scale bar: 10 µm). (I) Overall survival of the 57 CRC patients according to PVT1‐104aa expression detected by IHC score from Figure S2F. *p < .05.

Polysomal profiling through qPCR was employed to determine the ribosomal association of ectopically expressed circPVT1 in 293T cells across heavy (H), light (L) and monosome (M) fractions. Consistent with the positive control c‐Myc mRNA, circPVT1's presence in heavy and light polysomal fractions demonstrates its capacity for ribosome association and protein‐coding potential. The majority of linear PVT1 transcripts (negative control) predominantly localized to the monosomal (M) fraction, consistent with their expected ribosomal distribution pattern (Figure 2A). A circRNA‐specific reporter system verified IRES‐mediated translation in circPVT1, characteristic of the initiation mechanism shared by numerous translatable circular RNAs (Figure 2B; Figure S2A). A custom antibody targeting the unique C‐terminal peptide of the circPVT1‐encoded protein was developed, and its specificity was stringently verified by dot immunoblot analysis (Figure S2B). Immunoblotting specifically detected the endogenously encoded PVT1‐104aa along with its ectopically expressed linearized 3×Flag‐tagged version in 293T transfectants. ATG deletion in circPVT1 abolished PVT1‐104aa production despite maintained high circular RNA expression, confirming the translation dependence on this initiation site (Figure 2C,D; Figure S2C,D). By using anti‐Flag antibody and conducting immunoprecipitation‐mass spectrometry (IP‐MS), we confirmed the ∼18 kDa Flag‐tagged product (Figure S2E). In addition, endogenous PVT1‐104aa in Lovo cells was identified by mass spectrometry (MS) at the predicted molecular weight (Figure 2E). Furthermore, the expression level of PVT1‐104aa protein is also regulated by PVT1 mRNA level (Figure S2F, upper and lower). Expression profiling of PVT1‐104aa was subsequently performed through comparative analysis of human intestinal epithelial controls and CRC‐derived cellular models. Consistent with circPVT1 expression, PVT1‐104aa levels showed marked elevation in CRC cells relative to normal intestinal epithelium, with particularly prominent expression in Lovo and SW620 lines (Figure 2F). Clinical specimen analysis revealed universal detection of PVT1‐104aa in all randomly selected CRC tissues, with expression levels showing a marked increase compared with matched adjacent normal tissues (Figure 2G). Furthermore, we detected the expression level of PVT1‐104aa in CRC tissue samples at different stages. The results demonstrated that PVT1‐104aa expression increased with advancing disease stage, suggesting a close association between PVT1‐104aa expression levels and the malignancy of CRC (Figure S2G). Immunohistochemical (IHC) staining further confirmed that PVT1‐104aa was significantly upregulated in CRC tissues (Figure S2H). By conducting immunofluorescence with anti‐flag and anti‐PVT1‐104aa antibody, we confirmed that PVT1‐104aa localized in the nucleus in SW480 cells (Figure 2H). We further analyzed the impact of PVT1‐104aa expression on patient prognosis. Elevated PVT1‐104aa levels correlated significantly with adverse clinical outcomes, indicating its potential as a prognostic biomarker (Figure 2I). Collectively, these findings demonstrate circPVT1's protein‐coding capacity through PVT1‐104aa production, with elevated levels of this novel protein correlating with poorer patient survival in univariable analysis.

### 2.3 CircPVT1 is critical for proliferation and metastases of CRC cells

**FIGURE 3:**
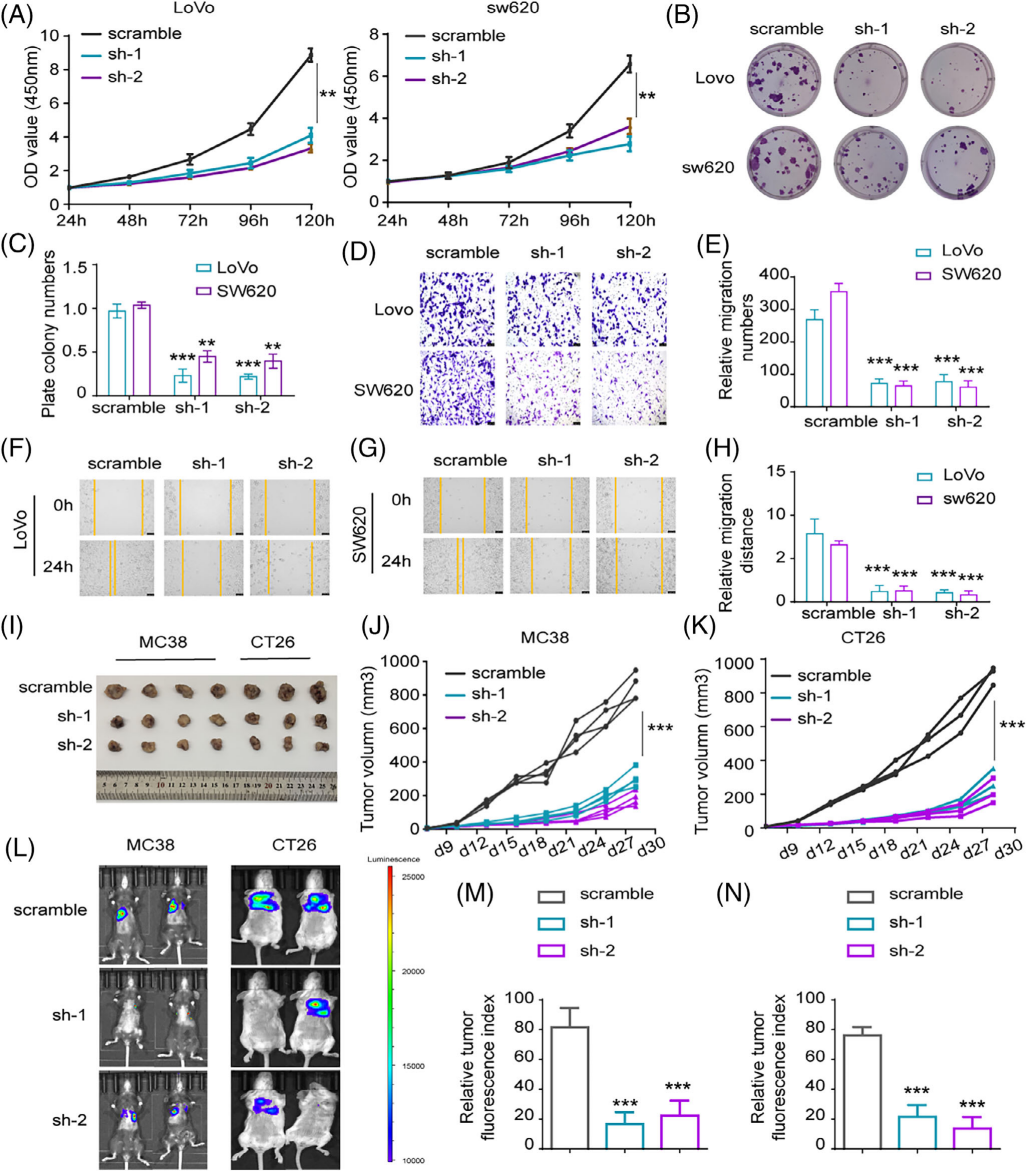
Oncogenic functions of circPVT1 in CRC cells. (A) CCK‐8 assay in Lovo and SW620 cells with stable circPVT1 knockdown (sh1/sh2) versus scramble control. (B, C) Colony formation capacity (B: representative images; C: quantification) of circPVT1‐depleted Lovo and SW620 cells. (D, E) Transwell migration assays (D: representative images; E: quantified colony numbers) showing impaired migration upon circPVT1 knockdown. (F–H) Wound healing assays; (F, G) representative images at 0/24 h; (H) quantified migration distance) demonstrating reduced motility in circPVT1‐silenced cells. (I–K) Xenograft tumour growth (I: excised tumours; J, K: growth curves) in mice implanted with MC38 or CT26 cells containing circPVT1 knockdown or control. (L–N) Lung metastasis models (L: bioluminescent images; M, N: quantified metastatic burden) showing reduced metastasis from circPVT1‐depleted MC38 cells. **p < .01, ***p < .001. *N* = 4 in each in vivo experiment.

To elucidate the functional significance of circPVT1 in colorectal carcinogenesis, we established stable circPVT1‐knockdown cell lines in Lovo and SW620 cells and verified the knockdown efficiency through qPCR and Western blot assays (Figure S3A,B). Growth suppression was observed in Lovo and SW620 cells following circPVT1 knockdown, as quantified by CCK‐8 assays (Figure 3A). Consistent with the proliferation data, circPVT1 silencing significantly diminished colony‐forming efficiency, underscoring its fundamental role in supporting CRC cell growth (Figure 3B,C). Transwell migration and wound healing assays further confirmed that circPVT1 knockdown significantly impaired the metastatic potential of colorectal cancer cells compared with control groups (Figure 3D–H). To further validate the biological function of circPVT1 in CRC under in vivo conditions, we established circPVT1 knockdown in both MC38 and CT26 cell lines and confirmed the knockdown efficiency using qPCR and Western blot analysis (Figure S3C,D). In the syngeneic tumour models (*N *= 4 in each in vivo experiment), circPVT1 knockdown significantly attenuated the tumour‐forming capacity of both MC38 and CT26 cells (Figure 3I–K; Figure S3E). An experimental lung metastasis model was established through tail vein administration of tumour cells (*N *= 4 in each in vivo experiment). CircPVT1‐knockdown cells demonstrated significantly diminished metastatic burden in lungs relative to controls (Figure 3L–N). Collectively, these data demonstrated that circPVT1 plays crucial oncogenic roles in CRC by promoting proliferation and metastatic potential.

### 2.4 PVT1‐104aa, instead of circ‐PVT1, exerts the pro‐tumour functions

**FIGURE 4:**
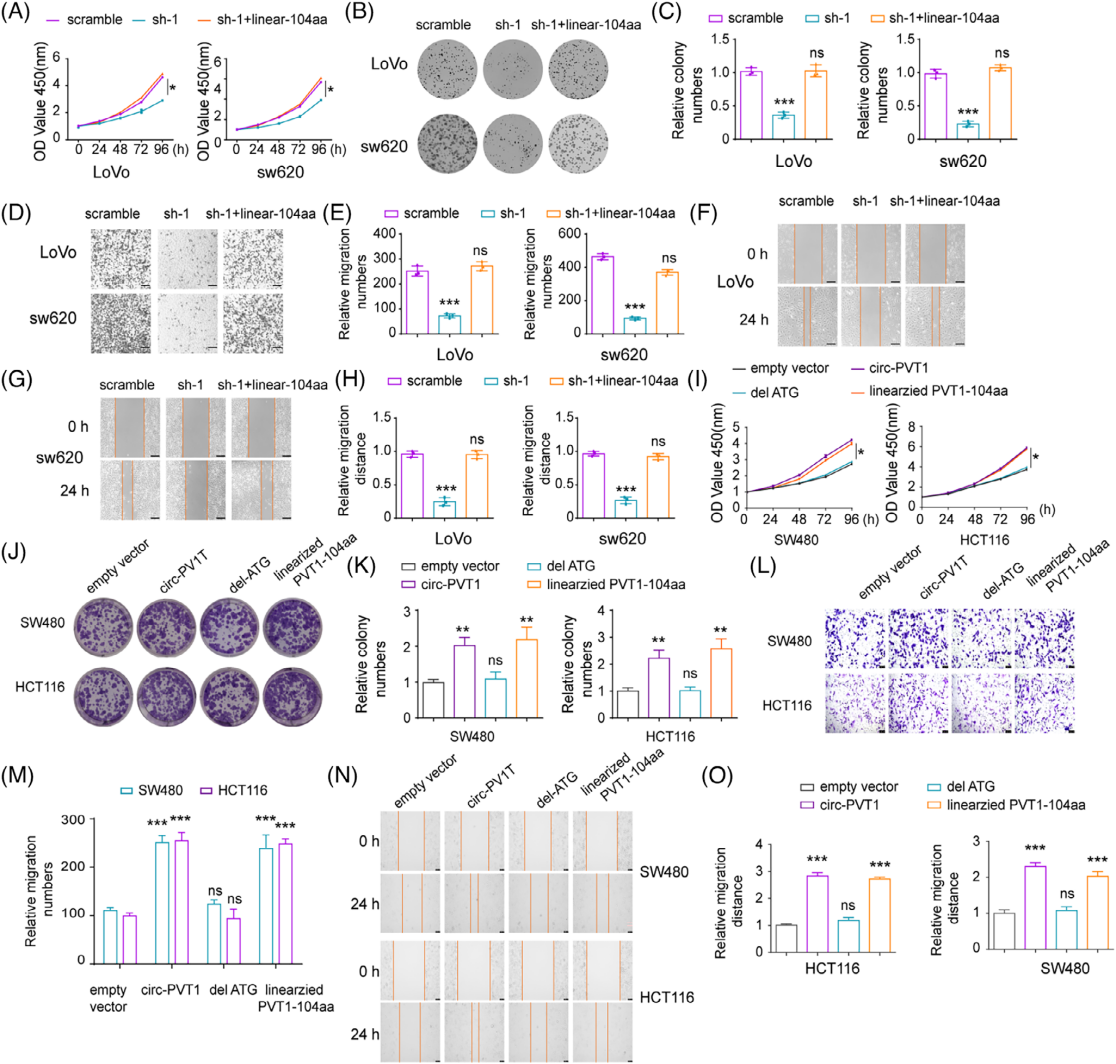
PVT1‐104aa mediates the oncogenic functions of circPVT1 in CRC. (A) CCK‐8 proliferation assay in Lovo and SW620 cells with: (1) scramble control, (2) circPVT1 shRNA (sh1), or (3) sh1 + PVT1‐104aa rescue. (B, C) Colony formation capacity (B: representative images; C: quantification) under the indicated conditions. (D, E) Transwell migration assays (D: representative images; E: quantified colonies). (F–H) Wound healing assays (F, G: representative images; H: migration distance quantification). (I) CCK‐8 proliferation in SW480/HCT116 cells expressing: (1) empty vector, (2) circPVT1, (3) ATG‐deleted circPVT1 (del‐ATG), or (4) PVT1‐104aa. (J, K) Colony formation (J: images; K: quantification) in SW480/HCT116 cells. (L, M) Transwell migration (L: images; M: colony counts). (N, O) Wound healing (N: images; O: distance of migration). **p < .01, ***p < .001.

To determine whether the oncogenic effects were mediated by circPVT1 or its encoded peptide, we performed rescue experiments by re‐expressing PVT1‐104aa in circPVT1‐knockdown CRC cells using a linearized PVT1‐104aa expression plasmid (western blotting confirmed successful transfection (Figure S3F,G). Notably, PVT1‐104aa restoration completely rescued the malignant phenotypes attenuated by circPVT1 knockdown (Figure 4A–H). To exclude potential structural effects of circPVT1 independent of translation, we examined the del‐ATG circPVT1 mutant, which cannot produce PVT1‐104aa. Functional assays demonstrated that del‐ATG circPVT1 failed to enhance proliferation or invasion in CRC cells (Figure 4I–O), further confirming that the tumour‐promoting effects specifically require PVT1‐104aa translation rather than circPVT1 RNA itself. These data establish the encoded product PVT1‐104aa, not the parental circPVT1, as the functional tumour promoter.

### 2.5 PVT1‐104aa enhances MYC expression by inhibiting its degradation

**FIGURE 5:**
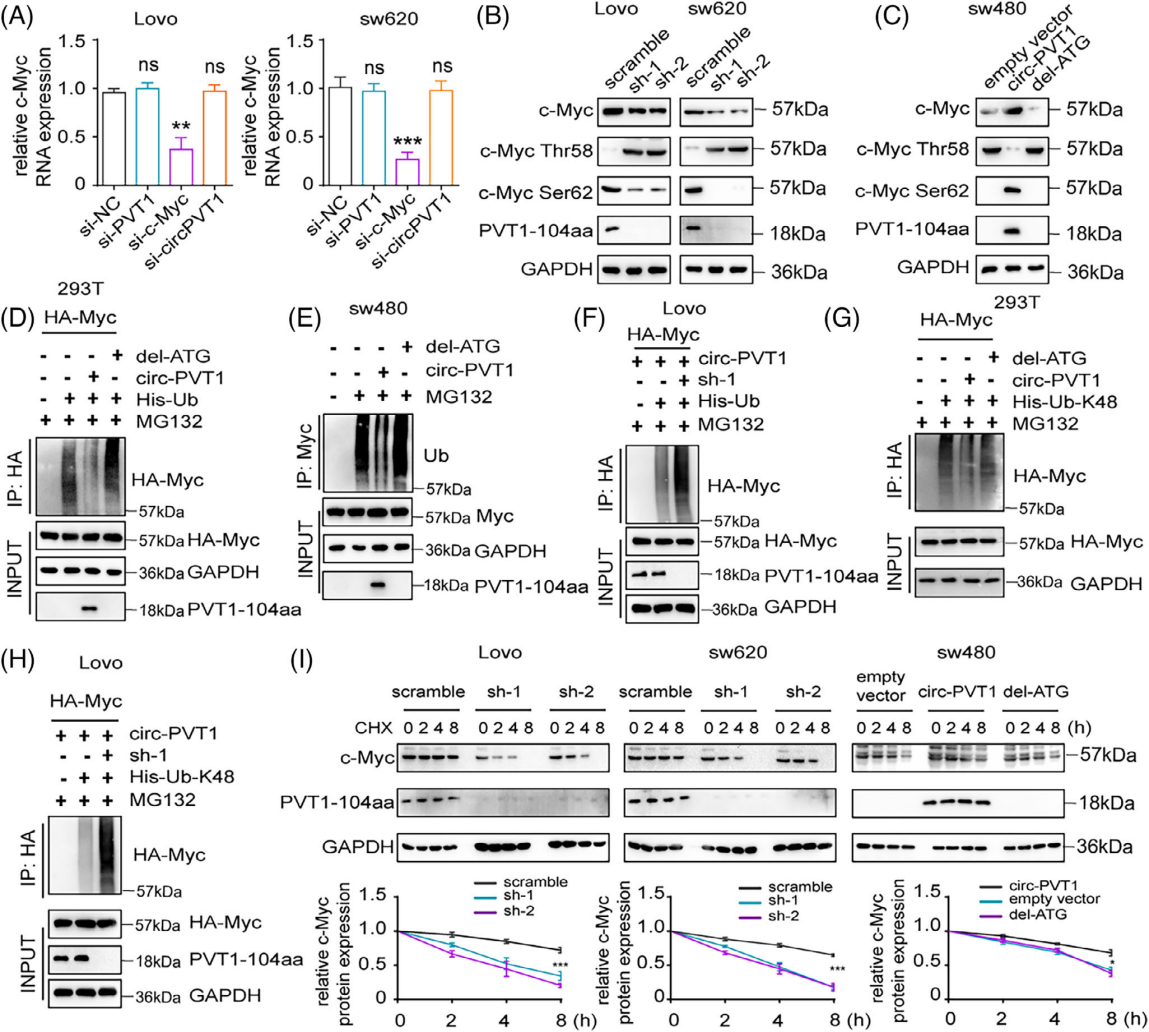
PVT1‐104aa stabilizes MYC protein by impeding degradation by ubiquitination. (A) c‐Myc mRNA levels were detected in Lovo and SW620 cells transfected with PVT1, c‐Myc, circPVT1 siRNAs or control cells by qPCR. (B) Western blotting analysis showed the c‐Myc protein level in Lovo or SW620 cells, which were transiently transfected with either sh‐circ‐PVT1 or scrambled cells. (C) Western blotting was performed on WCL from SW480 cells transfected with either a control vector, circ‐PVT1‐expressing vector, or del‐ATG vector. (D) Immunoprecipitation (IP) of HA‐tagged proteins was detected in 293T cells transfected with the control vector, circ‐PVT1‐expressing vector, or del‐ATG mutant vector, following a 12 h MG132 (10 µmol/L) treatment. (E) WCL and Ub pull‐down samples from SW480 cells (transfected as indicated and treated with 10 µmol/L MG132 for 12 h) were subjected to western blotting. (F) HA‐tagged proteins were pulled down from Lovo cells with indicated shRNAs or constructs, following a 12 h pretreatment with 10 µmol/L MG132. (G) Immunoprecipitation using HA antibody was performed in 293T cells with circ‐PVT1 or del‐ATG transfection after a 12 h MG132 (10 µM) treatment. (H) HA pull‐down assays were conducted in Lovo cells expressing sh‐circ‐PVT1 or control constructs, following a 12 h MG132 (10 µmol/L) treatment. I MYC protein half‐life analysis in Lovo, SW620 cells that were transfected with scrambled, sh‐1 or sh2 as mentioned above, and in SW480 cells transfected with plasmids expressing control vector, circ‐PVT1 or del‐ATG vector. c‐Myc band density relative to GAPDH was quantified and plotted as indicated.

Due to the close relationship between PVT1 mRNA level and c‐Myc expression, we naturally explored the association between PVT1‐104aa and c‐Myc expression. qPCR analysis revealed that neither siPVT1 nor si‐circPVT1 significantly altered mRNA levels of c‐Myc in CRC cells (Figure 5A). However, in both Lovo and SW620 cells, the expression level of c‐Myc protein was significantly reduced following the knockdown of circPVT1 (Figure 5B). In contrast, circPVT1 overexpression increased c‐Myc protein expression. Modification of circPVT1 del‐ATG expression increased circPVT1 RNA levels but did not alter c‐Myc protein or mRNA levels, which further supported that it was PVT1‐104aa, but not circPVT1, that regulated c‐Myc protein expression (Figure 5C; Figure S4A). Furthermore, in non‐Myc‐driven CRC cell lines (DLD1 and HCT15), the overexpression of circPVT1 also led to a significant increase in c‐Myc expression levels (Figure S4B). To explore the pathways that PVT1‐104aa regulates c‐Myc protein abundance, Lovo cells with circPVT1 knockdown were treated with MG132 (proteasome inhibitor), NH_4_Cl (lysosome inhibitor) and 3‐MA (autophagy inhibitor 3‐methyladenine), respectively. The results manifested that MG132 could obviously impede the degradation of c‐Myc but not NH_4_Cl or 3‐MA, which indicated that PVT1‐104aa may regulate c‐Myc abundance through protein degradation pathways (Figure S4C). The ubiquitin‐proteasome system (UPS) represents the predominant pathway mediating c‐Myc protein degradation. Then we overexpressed circPVT1 (del‐ATG as control) to detect the c‐Myc ubiquitin levels in exogenous and endogenous conditions, respectively. The results showed that overexpression of circPVT1, but not the del‐ATG, inhibited the c‐Myc ubiquitination both in exogenous and endogenous (Figure 5D,E). In contrast, c‐Myc ubiquitination was improved under circPVT1 knockdown in Lovo and SW620 cells (Figure 5F; Figure S4D). In addition, all lysine residues of ubiquitin can be modified through K48, K63, or others. K48‐linked ubiquitin was associated with target protein degradation. Overexpression of circPVT1, but not del‐ATG, significantly reduced the expression level of c‐Myc K48‐linked ubiquitination, whereas knockdown of circPVT1 suppressed c‐Myc K48 levels (Figure 5G,H; Figure S4E,F). Furthermore, we investigated the relationship between c‐Myc protein half‐life and PVT1‐104aa expression levels. The results demonstrated that knockdown of circPVT1 markedly shortened the half‐life of c‐Myc, while overexpression of circPVT1 (but not del‐ATG) significantly prolonged c‐Myc stability (Figure 5I). These results indicate that PVT1‐104aa enhances c‐Myc expression levels by suppressing its ubiquitination‐mediated degradation.

### 2.6 PVT1‐104aa hinders the interaction between c‐Myc and GSK3β

**FIGURE 6:**
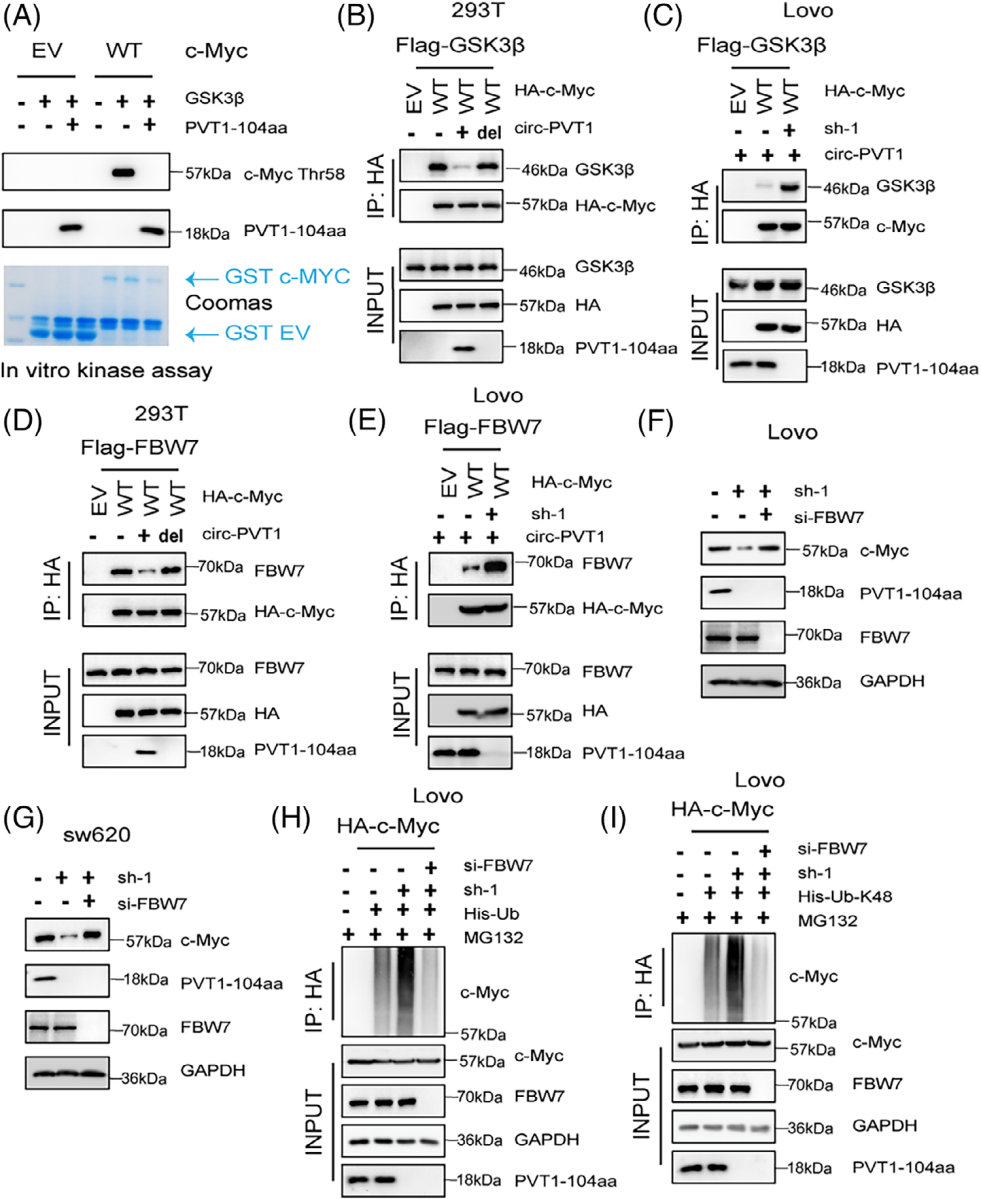
PVT1‐104aa hinders the interaction between MYC and GSK3β. (A) Kinase assays were conducted in vitro using purified GSK3β proteins and PVT1‐104aa, and purified GST c‐Myc in bacteria as substrate. (B) Western blot analysis showed the pull‐down proteins from 293T cells transfected with either a control vector, a circ‐PVT1‐expressing vector, a del‐ATG mutant vector, or other specified constructs. (C) Western blot analysis was performed on WCL and HA pull‐down samples derived from LoVo cells transfected with plasmids expressing control vector, sh‐circPVT1 and indicated constructs. (D) Western blot analysis was performed on WCL and HA pull‐down samples in 293T cells with control vector, circPVT1 or del‐ATG vector and indicated constructs. (E) WCL and HA‐IP samples from LoVo cells transfected with control, sh‐circPVT1, or other indicated plasmids were detected by immunoblotting. (F) Western blot analysis in LoVo cells transfected with sh‐circPVT1 or si‐FBW7 as indicated. (G) Western blot analysis in SW620 cells transfected with sh‐circPVT1 or si‐FBW7 as indicated. (H) IP analysis of Flag pulldown elements in LoVo cells transfected with the indicated plasmids and His‐Ub. MG132 (10 µmol/L) was treated for 12 h before cells were harvested. (I) IP analysis of Flag pulldown samples in LoVo cells transfected with His‐Ub‐K48 and indicated constructs. To inhibit proteasomal degradation, MG132 (10 µmol/L) was used to treat the cells for 12 h before the cells were harvested.

The ubiquitination level of c‐Myc is closely related to its phosphorylation level. Specifically, GSK3β facilitates c‐Myc phosphorylation on Thr58, resulting in the FBW7‐mediated destabilization of c‐Myc.^18^ Therefore, we speculate whether PVT1‐104aa affects protein content of c‐Myc via GSK3β and FBW7. Previous results showed that a high level of PVT1‐104aa expression inhibits c‐Myc Thr58, whereas it enhances c‐Myc Ser62 (Figure 5B,C). First, we found that PVT1‐104aa can directly inhibit the phosphorylation level of c‐Myc in vitro (Figure 6A). Then we tested the potential interaction between PVT1‐104aa and c‐Myc. 293T cells co‐transfected with HA‐tagged c‐Myc (HA‐Myc) and circPVT1 expression vectors confirmed their reciprocal binding through immunoprecipitation (IP) analysis, indicating the molecular interaction between PVT1‐104aa and c‐Myc (Figure S4G,H). To map PVT1‐104aa's binding interface on c‐Myc, we designed three truncation mutants spanning the N‐terminal domain (NTD, 1–143), central region (144‐320) and C‐terminal domain (CTD, 321–439). IP experiment confirmed specific interaction between PVT1‐104aa and c‐Myc's NTD (Figure S4I). To further explore whether PVT1‐104aa could affect the binding of c‐Myc and GSK3β, 293T cells transfected with Flag‐ GSK3β and HA‐c‐Myc, followed by an IP experiment, indicated that PVT1‐104aa remarkably disrupted c‐Myc‐GSK3β interaction (Figure 6B). Furthermore, knockdown of circPVT1 facilitated c‐Myc‐GSK3β interaction (Figure 6C). Previous studies have reported that FBW7 was a ubiquitin ligase of E3 for c‐Myc,^19^ we speculated that PVT1‐104aa may also influence the binding of FBW7 with c‐Myc. We confirmed our prediction again that c‐Myc‐FBW7 interaction was inhibited when PVT1‐104aa overexpression and facilitated when circ‐PVT1 knockdown (Figure 6D,E). To determine whether PVT1‐104aa regulates c‐Myc stability relies on the existence of FBW7, we performed FBW7 knockdown in circPVT1‐deficient Lovo and SW620 cells. Strikingly, FBW7 depletion abrogated the decline of c‐Myc caused by circPVT1 silencing, restoring c‐Myc expression to baseline levels (Figure 6F,G). IP experiment further verified that knockdown of FBW7 would decrease c‐Myc ubiquitination levels in circPVT1 knockdown CRC cell lines (Figure 6H,I). Taken together, we demonstrate that PVT1‐104aa promotes c‐Myc expression, which depends on GSK3β‐mediated phosphorylation and FBW7‐mediated ubiquitination.

### 2.7 PVT1‐104aa regulates PD‐L1 expression in CRC cells

**FIGURE 7:**
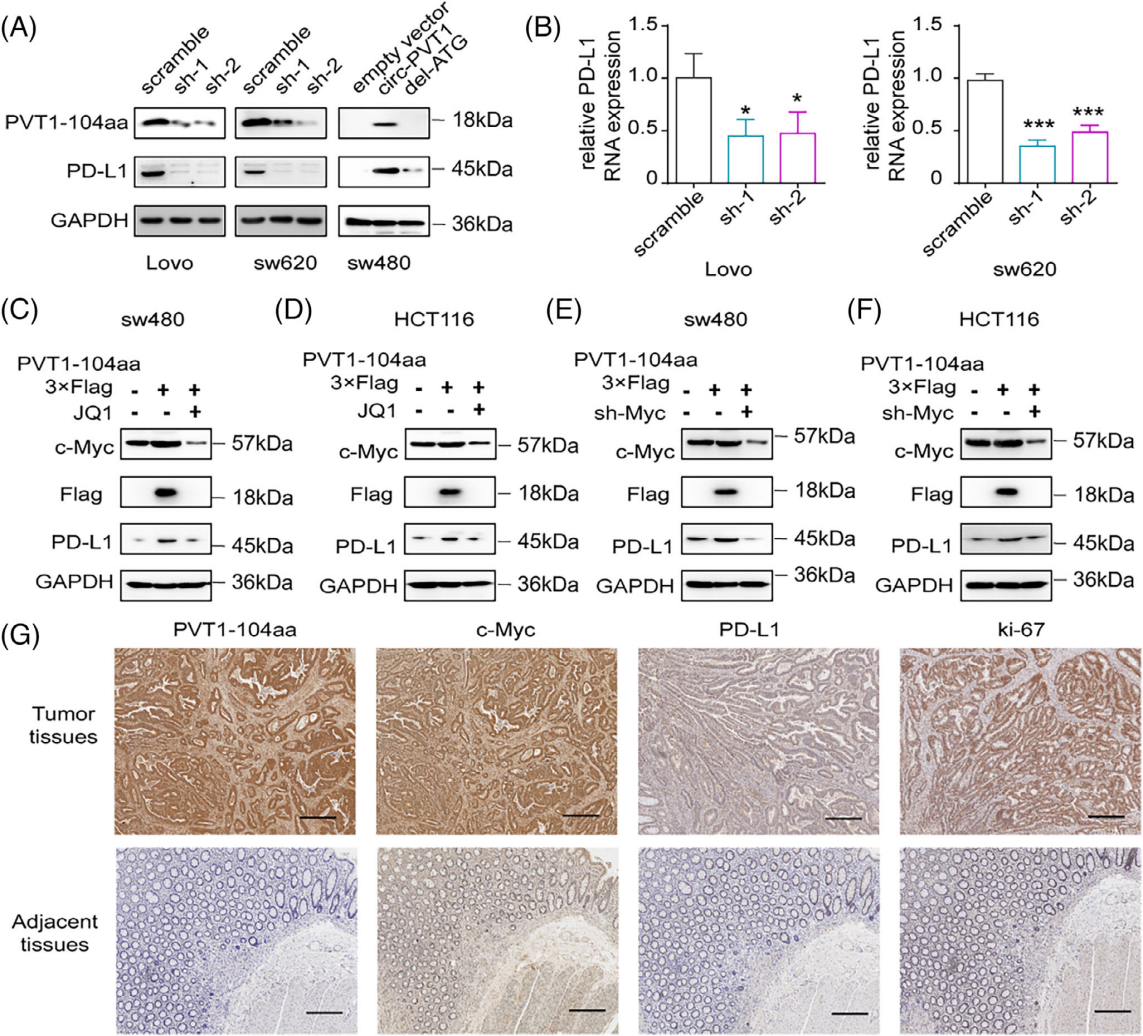
PVT1‐104aa regulates PD‐L1 expression in CRC cells through Myc. (A) Western blot analysis of LoVo, SW620 and SW480 cells transfected with shRNAs (control, sh‐1, sh‐2) or plasmids (control, circPVT1, del‐ATG) with the indicated antibodies. (B) qPCR analysis of PD‐L1 mRNA levels in circPVT1‐depleted LoVo/SW620 cells. (C) IB analysis of SW480 cells transfected or treated with linearized PVT1‐104aa or JQ1, as indicated, with antibodies as shown above. (D) IB analysis of HCT‐116 cells transfected or treated with linearized PVT1‐104aa or JQ1, as indicated, with antibodies as shown above. (E) IB analysis of SW480 cells transfected with linearized PVT1‐104aa or c‐Myc shRNA with the indicated antibodies. (F) IB analysis of HCT‐116 cells transfected with linearized PVT1‐104aa or c‐Myc shRNA with the indicated antibodies. (G) Representative IHC staining showing correlated expression of MYC, PD‐L1 and PVT1‐104aa in CRC specimens (scale bars: 250 µm).

It has been found that the expression level of c‐Myc is positively correlated with that of PD‐L1 in various cancer cells.^20^, ^21^ Using promoter reporter assays and ChIP‐qPCR, we first validated that c‐Myc transcriptionally regulates PD‐L1 expression by directly binding to its promoter region (Figure S4J,K); moreover, c‐Myc‐mediated transcriptional regulation of PD‐L1 was positively correlated with PVT1‐104aa expression levels (Figure S4L–O). Based on these findings, we hypothesized that PVT1‐104aa might also regulate PD‐L1 expression levels. In Lovo and SW620 cells, knockdown of circPVT1 significantly reduced PD‐L1 expression levels. Conversely, in SW480 cells, overexpression of circPVT1 (but not del‐ATG) markedly upregulated PD‐L1 expression, which further demonstrates that PVT1‐104aa, rather than the circPVT1 RNA itself, modulates PD‐L1 expression levels (Figure 7A). Consistent with these findings, reduced PVT1‐104aa expression led to decreased PD‐L1 mRNA levels (Figure 7B). In addition, pharmacological inhibition of c‐Myc using JQ1 or genetic silencing of c‐Myc expression abrogated the PVT1‐104aa‐induced upregulation of PD‐L1, demonstrating c‐Myc's essential role in this regulatory axis (Figure 7C–F). Moreover, immunohistochemical analysis of clinical specimens revealed significant positive correlations between PVT1‐104aa expression levels and both c‐Myc and PD‐L1 protein expression (Figure 7G). Collectively, these results demonstrate that PVT1‐104aa regulates PD‐L1 expression through a c‐Myc‐dependent mechanism.

### 2.8 PVT1‐104aa is a promising therapeutic target of CRC

**FIGURE 8:**
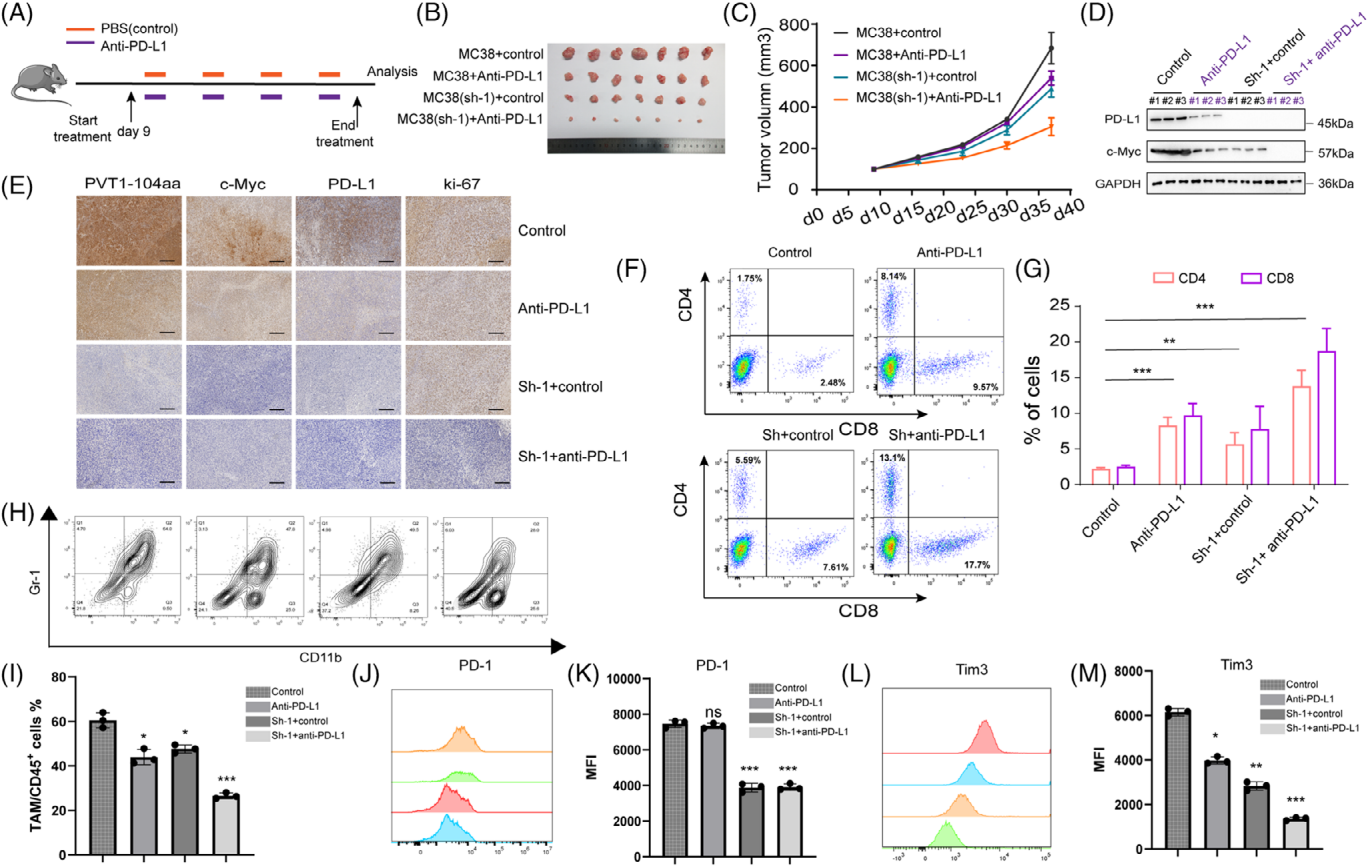
PVT1‐104aa is a promising therapeutic target of CRC. (A) Treatment scheme: C57BL/6 mice with established allografts received: vehicle control or anti‐PD‐L1 (100 µg/injection, twice weekly for 4 cycles). (B) Representative images of tumours from the four groups with different treatments as indicated. (C) Tumour growth curve of the four groups with different treatments. (D) Protein lysates from xenograft tumours across all four treatment cohorts were analyzed by immunoblotting using antibodies as indicated. (E) Representative IHC staining of MYC, PD‐L1, Ki‐67 and PVT1‐104aa in xenografts (scale bars: 100 µm). (F, G) Flow cytometry analysis of TILs: CD4^+^ and CD8^+^ T cell populations across different treatment groups. All in vivo experiments used *n* = 7 mice/group. Source data are provided. (H) Representative flow cytometry contour plots of CD11b and Gr‐1 expression in tumour‐infiltrating immune cells across four groups: Control, anti‐PD‐L1, Sh‐1+Control and Sh‐1+anti–PD‐L1. (I) Quantification of CD11b^+^Gr‐1^+^ MDSCs among CD45^+^ tumour‐associated myeloid cells. (J, K) Histograms showing PD‐1 expression on CD8^+^ T cells from each group and corresponding MFI quantification. (L, M) Histograms and quantification of Tim‐3 expression on CD8^+^ T cells, shown as MFI. *p < .05, **p < .01, ***p < .001. *N* = 7 in each in vivo experiment.

The above findings prompted us to investigate the relationship between PVT1‐104aa expression levels and PD‐L1 inhibitor efficacy in a murine CRC model. We developed syngeneic tumour models in C57BL/6 mice using both wild‐type MC38 cells and circPVT1‐knockdown MC38 cells to investigate the effect of PVT1‐104aa expression levels on PD‐L1 inhibitor efficacy, with comparison to placebo controls (*N *= 7 in each in vivo experiment). Anti‐PD‐L1 antibody was administered intraperitoneally at 100 µg per dose per mouse twice weekly for four consecutive weeks (Figure 8A). The results demonstrate that while either PVT1‐104aa knockdown alone or PD‐L1 inhibitor monotherapy modestly suppressed tumour growth in vivo, the combination treatment exerted significantly enhanced antitumour effects, revealing a synergistic interaction between these two modalities (Figure 8B,C; Figure 5A,B). Compared with the other three treatment groups, tumours receiving combined PVT1‐104aa knockdown and PD‐L1 inhibitor therapy exhibited the lowest expression levels of both c‐Myc and PD‐L1 (Figure 8D,E). The c‐Myc and PD‐L1 also decreased in xenograft tumours of the sh‐1 and sh‐2 groups compared with the scramble group, as described in Figure 3I (Figure S5C,D). To assess the effects of PVT1‐104aa depletion and anti‐PD‐L1 therapy on the tumour microenvironment, we tested the tumour‐infiltrating lymphocytes (TILs) in the above four treatment groups by flow cytometry. Comparative analysis revealed significantly enhanced infiltration of both CD4^+^ and CD8^+^ T cells in tumour tissues from the PVT1‐104aa knockdown group, the PD‐L1 inhibitor monotherapy group and the combination therapy groups relative to controls. The most pronounced effect was observed in the combination groups (Figure 8F,G). Furthermore, we also detected an increase in CD4^+^ and CD8^+^ T cells infiltration in xenograft tumours (from Figure 3I) with PVT1‐104aa deprivation (Figure S5E). To evaluate the effects of PVT1‐104aa knockdown and anti–PD‐L1 treatment on tumour‐associated myeloid‐derived suppressor cells (MDSCs) and T cell exhaustion, we analyzed TILs by flow cytometry. Compared with the control group, the proportion of CD11b^+^Gr‐1^+^ MDSCs within CD45^+^ tumour‐associated immune cells was significantly lower in the PVT1‐104aa knockdown group, with a further reduction observed when Sh‐1 knockdown was combined with anti‐PD‐L1 therapy. Quantitative analysis revealed a synergistic decrease in MDSCs in the PVT1‐104aa knockdown plus anti‐PD‐L1 treatment group (Figure 8H,I). To further assess T‐cell function, we quantified the expression of the cytotoxic markers Granzyme B (GZMB) and Perforin (PRF1) in tumour‐infiltrating T cells by flow cytometry. The positive rates for these markers across the experimental groups are presented in Figure S5F. Additionally, the expression levels of exhaustion markers PD‐1 and Tim‐3 on CD8^+^ T cells, as measured by mean fluorescence intensity (MFI), were markedly reduced in the PVT1‐104aa knockdown group and were even further suppressed in the combination treatment group (Figure 8J–M). Collectively, these experimental results establish PVT1‐104aa as a promising therapeutic target in CRC. The combined strategy of PVT1‐104aa inhibition and PD‐L1 blockade demonstrates significant synergistic efficacy in murine CRC models, suggesting a novel combinatorial approach for clinical translation.

## 3 DISCUSSION

The emergence of immune checkpoint inhibitors (ICIs) has revolutionized cancer therapeutics, ushering in a new era of tumour immunotherapy. While ICIs have demonstrated remarkable efficacy across multiple malignancies, their clinical benefits in CRC remain predominantly restricted to the microsatellite instability‐high (MSI‐H) subtype, which represents merely 5% of recurrent or metastatic cases.^22^, ^23^ This striking limitation underscores the persistent therapeutic challenges in managing microsatellite‐stable (MSS) CRC, which constitutes the majority (95%) of metastatic cases. Consequently, overcoming primary resistance and converting ICI‐refractory MSS tumours into ICI‐responsive malignancies has emerged as a critical research priority in colorectal oncology.

Initially, circRNAs were widely regarded as non‐coding RNAs.^24^ However, emerging studies have demonstrated that certain circRNAs can be translated into functional proteins.^25^, ^26^, ^27^ Notably, all previously reported translatable circRNAs were derived from protein‐coding parent genes, with no documented cases of circRNAs originating from non‐coding genes exhibiting translational potential. In this study, we report a novel finding: The circRNA derived from the non‐coding gene *PVT1* (designated circPVT1) encodes a functional 104‐amino acid peptide (PVT1‐104aa). Importantly, we demonstrate that PVT1‐104aa functionally promotes CRC progression by enhancing both proliferative and metastatic capacities.

Oncogenic c‐Myc elevation, frequently driven by *MYC* amplification or transcriptional activation, represents a well‐established pathogenetic mechanism across diverse malignancies.^28^ Among well‐characterized solid tumour models such as hepatocellular and mammary carcinomas, *MYC* gene amplification is frequently observed.^29^, ^30^ In contrast, chromosomal translocation of *MYC* predominantly occurs in B‐cell or T‐cell lymphomas and leukaemias.^31^ Despite substantial evidence demonstrating the critical oncogenic role of c‐Myc in tumorigenesis and cancer progression, its intrinsically disordered protein structure has rendered it a challenging therapeutic target for conventional drug development.^32^ However, emerging technologies such as proteolysis targeting chimaeras (PROTACs) offer promising strategies for targeting c‐Myc,^33^, ^34^ potentially enabling the future development of effective c‐Myc‐directed therapies.

The *MYC* gene is located at chromosomal region 8q24.21, a locus frequently amplified in colorectal cancer. Notably, within the 8q24.21 region, *MYC* is virtually the only protein‐coding gene surrounded by predominantly non‐coding genes, rendering this genomic area a characteristic ‘gene desert’. Of particular significance, co‐amplification of *MYC* and its adjacent gene *PVT1* has been demonstrated to induce tumour formation in mouse models. However, genetic ablation of *PVT1* reveals that *MYC* amplification alone is insufficient for tumorigenesis, indicating an essential cooperative role of *PVT1* in *MYC*‐driven oncogenesis.^9^ In this study, we demonstrate that PVT1‐104aa inhibits ubiquitin‐mediated degradation of c‐Myc, thereby enhancing and sustaining its protein expression levels. Furthermore, PVT1‐104aa expression is positively regulated by the *PVT1* gene. These findings establish a novel mechanistic basis for *PVT1*‐mediated regulation of c‐Myc expression.

Transcriptional control of PD‐L1 is directly mediated through c‐Myc binding to its promoter regulatory elements.^20^ Beyond its established role in tumorigenesis, c‐Myc contributes to immunosuppression and facilitates tumour immune evasion.^35^ Our study further demonstrates a positive correlation between PVT1‐104aa, c‐Myc and PD‐L1 expression levels. Notably, in CRC cells, knockdown of PVT1‐104aa significantly reduces both c‐Myc and PD‐L1 expression. Moreover, in vivo experiments reveal that combined inhibition of PVT1‐104aa and anti‐PD‐L1 therapy exhibits marked synergistic effects in suppressing tumour growth. While prior studies have established that a specific PD‐L1^+^ CD206^+^ macrophage subpopulation is critical for effective immunotherapy in the colorectal cancer immune microenvironment,^36^ it remains to be elucidated whether the observed synergistic antitumour effect is dependent on other PD‐L1^+^ cell types beyond CD4^+^ and CD8^+^ T cells. These findings provide novel insights into potential therapeutic strategies targeting CRC progression and immune escape mechanisms.

### 3.1 Limitations

Our study has several limitations that warrant consideration. Notably, substantial differences exist between murine and human immune systems, including disparities in immune cell populations, antibody responses and cytokine profiles. These interspecies variations may affect the translational relevance of our findings. To address this, future studies using humanized immune system mouse models would provide more clinically predictive data to evaluate the combined therapeutic efficacy of PVT1‐104aa inhibition and anti‐PD‐L1 blockade in CRC. On the other hand, while our findings demonstrate the oncogenic role of PVT1‐104aa in CRC, its therapeutic targeting warrants careful evaluation of potential off‐target effects. To mitigate such risks, future studies should systematically assess PVT1‐104aa inhibition in normal cell lines to evaluate cytotoxicity.

## 4 CONCLUSIONS

Our study identified PVT1‐104aa as a novel oncogenic protein that is significantly upregulated in CRC tissues and associated with poor patient prognosis. Mechanistically, PVT1‐104aa regulates PD‐L1 expression through c‐Myc, thereby promoting tumour immune evasion. Importantly, in vivo experiments demonstrated that targeting PVT1‐104aa synergizes with anti‐PD‐L1 therapy, offering a promising combinatorial strategy for CRC treatment. These findings establish PVT1‐104aa as both a prognostic biomarker and a therapeutic target in CRC. In addition, PROTAC drugs can be developed in the future to achieve targeted degradation of PVT1‐104aa,^33^, ^34^ so as to achieve the purpose of disease treatment.

## 5 MATERIALS AND METHODS

### 5.1 Human tissues

Following informed consent acquisition, matched colorectal carcinoma and histologically normal adjacent tissue specimens were surgically collected from patients treated at Fudan University Shanghai Cancer Center. Histopathological evaluation of all specimens was performed by certified pathologists.

### 5.2 Cell lines

The HEK293T cells and CRC cell lines, including HCT116, Lovo, RKO, SW480, SW620 and DLD1, were procured through the ATCC repository in Manassas, Virginia. NCM460 human normal colonic epithelial cells (cat. no. IM‐H445), murine colon carcinoma lines MC38 (cat. no. IM‐M006) and CT26 (cat. no. IM‐M007) were purchased from Immocell Biotechnology. Cellular propagation was conducted using DMEM (Gibco) as the basal medium, supplemented with 10% fetal bovine serum (Gibco) and 1% penicillin/streptomycin antibiotic solution, with incubation parameters set at 37°C/5% CO_2_ in a humidity‐controlled chamber. Before experimental use, all lines were confirmed mycoplasma‐negative and used within 10 passages post‐thaw.

### 5.3 RNA‐seq analysis

#### 5.3.1 RNA extraction and quality control

RNA extraction from samples was conducted with TRIzol reagent (Invitrogen, Carlsbad, CA, USA) in strict adherence to the manufacturer's specifications. RNA preparation quality was verified through electrophoretic analysis on RNase‐free agarose gels and quantitative assessment using a NanoDrop One spectrophotometer. RNA samples with RNA integrity numbers >7.0 and clear 18S/28S ribosomal RNA bands were used for downstream applications.

#### 5.3.2 cDNA library preparation

Following extraction, RNA was subjected to cDNA library construction. Given the focus on circular RNA (circRNA) analysis, ribosomal RNA depletion from total RNA was carried out with the Ribo‐off rRNA Depletion Kit (Vazyme, Cat. No. N406). To further enrich circRNAs, the rRNA‐depleted RNA was treated with RNase R (Takara, Cat. No. 2396) at 37°C for 15 min to degrade linear RNAs. Library generation was then conducted applying the TruSeq Stranded Total RNA Library Prep System (Illumina, Cat. No. 20020599). Briefly, the enriched RNA was fragmented using divalent cations under elevated temperature (94°C). cDNA synthesis was initiated with SuperScript IV Reverse Transcriptase (Thermo Fisher Scientific, Cat. No. 18090010) and random hexamers in the first step, followed by second‐strand generation using DNA Polymerase I and RNase H (NEBNext Second Strand Synthesis Module, NEB, Cat. No. E6111). The cDNA fragments underwent end repair and A‐tailing (NEBNext Ultra II End Repair/dA‐Tailing Module, NEB, Cat. No. E7546), followed by adapter ligation (TruSeq RNA UD Indexes, Illumina, Cat. No. 20022371). Finally, to prepare sequencing‐ready libraries, the products were subjected to size selection on E‐Gel™ EX Agarose Gels (Thermo Fisher Scientific, G402002), followed by PCR amplification with KAPA HiFi HotStart ReadyMix (Roche, KK2602).

#### 5.3.3 circRNA identification and quantification workflow

The primary bioinformatic workflow entailed quality assessment of raw sequencing reads by FastQC (v0.11.9) and subsequent genome alignment to the GRCh38 reference using STAR (v2.7.10a). Back‐splice junctions (BSJs) were identified using both CIRCexplorer3 (v3.0.0) and CIRI2 (v2.0.6), and only circRNAs detected by both tools were retained for downstream analysis to ensure high‐confidence calls. CircRNA quantification was obtained through feature Counts (v2.0.3), with differentially expressed circRNAs subsequently identified by edgeR (v3.40.0) using thresholds of FDR < .05 and |log2FC| > 1.

#### 5.3.4 Sequencing

All samples underwent deep transcriptome sequencing on an Illumina NovaSeq 6000 system configured for 150 bp paired‐end reads, employing NovaSeq 6000 S4 Reagent Kits (Cat. No. 20027465) with a target depth of 30 million reads per library to ensure comprehensive coverage.

#### 5.3.5 Bioinformatics analysis

Initial quality control of sequencing data involved a comprehensive evaluation with FastQC, with subsequent refinement through Trimmomatic for adapter contamination removal and low‐quality base trimming. Genome mapping of the processed reads to the human reference GRCh38 was conducted with the STAR aligner. Utilizing feature Counts for transcript quantification, we identified statistically significant genes through DESeq2 analysis in R, with criteria set at FDR < .05 and absolute log2 fold change exceeding 1. KEGG pathway enrichment analysis was systematically performed using target genes of differentially expressed miRNAs (upregulated or downregulated in each comparison group) as input gene sets. The GOstats package in R was employed to conduct statistical testing for gene enrichment across each functional GO term and KEGG pathway. A stringent threshold of *p*‐value < .01 was applied to determine significant pathway enrichment.

### 5.4 RNA qRT‐PCR

We synthesized complementary DNA with the HiScript III All‐in‐one RT SuperMix (Vazyme, #R333‐01), a kit specifically designed for qPCR applications. The CFX96 real‐time PCR system (Bio‐Rad) was used for all RT‐qPCR analyses using AceQ qPCR Probe Master Mix (Vazyme, #Q112‐02) as the detection chemistry. The specific experimental conditions for RT‐qPCR are as follows: Reverse transcription: 50°C for 15 min, 85°C for 5 s; qPCR: Stage 1 (initial denaturation) at 95°C for 5 min; Stage 2 (cycling reaction): 95°C for 10 s, 60°C for 30 s, repeated for 40 cycles. Following the primer details listed in Table S3, normalization against β‐actin and subsequent calculation of relative expression for both circRNAs and mRNAs were performed through the 2^−△△C^ *^t^* method. Each experiment was repeated in triplicate.

### 5.5 RNase R treatments

Validation of circPVT1's circular conformation involved enzymatic treatment of total RNA using RNase R (Lucigen) under standardized conditions (37°C, 15 min incubation), with molecular persistence evaluated through reverse transcription‐quantitative PCR.

### 5.6 Actinomycin D assay

Dactinomycin (2 µg/mL, MedChemExpress) was added to fifty thousand 293T cells for 0–24 h. RNA was harvested at the indicated timepoints for circ‐PVT1/linear PVT1 quantification by qPCR, normalized to 0 h controls.

### 5.7 Fluorescence in situ hybridization

We prepared Lovo cells for fluorescence in situ hybridization (FISH) by plating them on poly‐L‐ornithine‐coated coverslips (Sigma‐Aldrich) before hybridization. The procedure was carried out following the protocol of an RNA FISH kit (GenePharma). Image acquisition utilized a ZEISS LSM 880 microscope integrated with Airyscan super‐resolution technology, employing fluorescence in situ hybridization probes as specified in Table S2.

### 5.8 Sucrose gradient fractionation assay

#### 5.8.1 Sucrose gradient preparation

Before polysome isolation, 1 × 10^7^ cells in suspension received 100 µg/mL cycloheximide treatment (formulated in DMSO) during 5 min incubations maintained at 37°C. We lysed cells in 500 µL of polysome extraction buffer and centrifuged the lysates at 12 000×*g* for 10 min to remove cellular debris. The clarified lysate was carefully layered onto a discontinuous sucrose gradient (5–50% w/v) and subjected to ultracentrifugation (20 000×*g*, 2 h, 4°C) using a Beckman SW41 swinging‐bucket rotor. Gradient separation was carried out employing the BioComp Piston Gradient Fractionator (Model 152), with real‐time UV absorbance monitoring at 254 nm. Purified RNA from fractionation procedures was evaluated using RT‐qPCR methodology to define transcript allocation across ribosomal fractions.

#### 5.8.2 Polysomes analysis

Polysome analysis was performed by treating 1 × 10^7^ cells with 100 µg/mL cycloheximide (37°C, 5 min), lysing in 500 µL extraction buffer and centrifuging (12 000×*g*, 10 min). We layered the supernatant onto a 5–50% sucrose gradient before ultracentrifugation (20 000×*g*, 2 h, 4°C; SW41 rotor). Fractions were collected (BioComp Fractionator) with UV254 monitoring, followed by RNA extraction and RT‐qPCR analysis of polysomal distribution.

### 5.9 LC‐MS analysis

For subsequent proteomic analysis, total protein extracts were separated via SDS‐PAGE, and the band migrating at approximately 17 kDa was processed for enzymatic digestion. Next, a QExactive mass spectrometer was employed to analyze the extracted peptides (Thermo Fisher Scientific). Spectral matching was performed with the Mascot platform (Matrix Science) against the NCBI non‐redundant protein collection.

### 5.10 Proteomic analysis

For spectral library generation, a pooled sample comprising 5 µg protein from each specimen was processed into a peptide library, which was subsequently analyzed by LC‐MS. We applied the SWATH‐MS technique to accomplish quantitative profiling of each biological sample. Before analysis, SWATH quantitative data underwent median normalization and Log2 transformation. Proteins meeting the significance threshold (*p* < .01 by Student's *t*‐test) were classified as differentially abundant.

### 5.11 Immunofluorescence

Cells were seeded on coverslips for a night until they reach 50% confluency. Following an initial PBS wash series (3 × 5 min), cellular fixation was achieved using 4% paraformaldehyde during a 10 min incubation, followed by three successive 5 min rinses with phosphate‐buffered saline. Cellular permeabilization was accomplished through incubation with 0.1% Triton X‐100 for 10 min under ambient conditions, followed by three PBS washing cycles. Following blocking using 1% bovine serum albumin, cellular samples on coverslips received primary antibody incubations (1:1000 dilution) at 4°C for 16 h. Protect from light to prevent fluorophore quenching. After incubation, wash the cells with PBS three times, 5 min each, to remove unbound antibodies. Alexa Fluor‐conjugated secondary antibody (Invitrogen). Nuclear counterstaining was performed using DAPI, with subsequent image acquisition conducted via fluorescence microscopy.

### 5.12 Stable cell line generation

SW480 and HCT116 cells were transfected with the empty vector, circPVT1, delATG, or linearized PVT104aa plasmids using Lipofectamine 3000 (Invitrogen), following the manufacturer's protocol. Stable cell lines were selected by puromycin for seven days (2 µg/mL). For stable knockdown of circPVT1 in LoVo and SW620 cells, HEK293T cells received lentiviral constructs (shRNA/cDNA from Gene‐Pharma, Shanghai) along with psPAX2/pMD2G packaging plasmids (Addgene). Transduced cells were subsequently subjected to antibiotic selection using hygromycin (200 µg/mL) or puromycin (1 µg/mL) based on the viral vector's resistance marker.

### 5.13 Plasmids

All plasmids—including Circ‐PVT1 overexpression construct, circPVT1 del ATG, circPVT1‐3×Flag, linearized‐PVT1‐104aa, and PVT1‐104aa‐3×Flag—were constructed by chemical gene synthesis based on the pCDH‐CMV‐MCS‐EF1‐copGFP‐T2A‐Puro backbone and procured from Shanghai Generay Biotech Co., Ltd. pcDNA3‐Flag‐FBW7 and pcDNA3‐Flag‐GSK3β were cloned into mammalian expression pCDNA3‐Flag vectors. pcDNA3‐HA‐c‐Myc and truncated constructions were cloned into mammalian expression pCDNA3‐HA vectors. pCMV‐His‐Ub and pCMV‐His‐Ub‐K48 were only cloned into mammalian expression pCMV‐GST vectors. Luciferase reporter plasmids were generated by Genomeditech. The 67–203 bp IRES element and its deletion mutant were PCR‐amplified and cloned into the intercistronic region separating Rluc and Fluc reporter genes.

### 5.14 Dual‐luciferase reporter system

After being transfected with the IRES reporter for 48 h, cells were lysed and washed. Luciferase activities were measured sequentially (firefly followed by Renilla) using Dual‐Glo reagents, with IRES activity calculated as Fluc/Rluc ratio. A putative c‐Myc binding motif (ACATGTGTGT) located within the CD274 promoter region was inserted into the pGL3‐basic luciferase reporter vector (Promega) to generate the reporter construct. Lovo cells were plated in 24‐well plates, followed by Lipofectamine 3000‐based transfection (Invitrogen) with 400 ng reporter construct and 40 ng pRL‐TK Renilla control vector per well. Following the 24 h incubation period, both firefly and Renilla luciferase signals were quantified with the dual‐luciferase reporter assay system (Promega) per the manufacturer's instructions. Normalization was performed by dividing firefly luciferase readings by the corresponding Renilla values, with results presented as relative luciferase units.

### 5.15 CCK8 assay

For cell proliferation assessment, cells were seeded at a density of 2 × 10^3^ cells per well in 96‐well plates, with each well containing 100 µL of complete medium, followed by overnight incubation (37°C, 5% CO_2_). On consecutive days (days 1–5), 10 µL test compounds were administered to each well, with subsequent 2 h incubations under standard culture conditions. Quantification was performed by measuring the optical density (OD) with a microplate reader, with all experiments conducted in triplicate.

### 5.16 Colony formation assay

For the colony formation assay, 2000 cells were seeded per well in six‐well plates and cultured for 14 days under standard conditions (37°C, 5% CO_2_), with the medium being refreshed every 72 h. After the 14‐day culture, cell colonies were fixed using 4% methanol (15 min, RT) followed by three 5 min PBS washes. Colonies then underwent staining using 0.1% crystal violet solution for 20 min at room temperature. After staining, colony images were acquired and quantified for analysis. Each experiment was repeated in triplicate.

### 5.17 Migration transwell assay

Cells (3 × 10^4^) in 200 µL serum‐free DMEM were placed in the upper transwell chamber of a 24‐well plate (Falcon #353097).To create a chemotactic gradient, the lower chamber contained 800 µL of 10% FBS‐supplemented DMEM. After the 24 h incubation under standard conditions (37°C, 5% CO_2_), cells that had migrated to the membrane's lower side were fixed in 100% methanol for 10 min at room temperature. After three PBS washes (5 min each), a 0.1% crystal violet solution was applied for 20 min. The stained migratory cells were then visualized using an inverted microscope at 100× magnification and quantified manually. Each experiment was repeated in triplicate.

### 5.18 Wound healing assay

For the wound healing assay, 1 × 10^5^ cells/well were seeded in six‐well plates and cultured overnight to form confluent monolayers in complete medium (DMEM+10% FBS). The medium was replaced with low‐serum medium (2% FBS), and a uniform scratch was created using a sterile 200 µL pipette tip, followed by PBS washes to remove debris. Wound closure was monitored by capturing phase‐contrast images at identical positions at 0, 12 and 24 h (100× magnification). Migration rates were quantified by measuring wound width changes using ImageJ software, calculated as:((0 h width—*t* h width)/0 h width)×100%, with triplicate wells per condition. Each experiment was repeated in triplicate.

### 5.19 Purification of GST‐tagged proteins from bacteria

The pGEX‐4T1‐GSK‐3β construct was transformed into BL21 (DE3) *E. coli* to express the GSK‐3β‐GST fusion protein. Following a 1:100 dilution of starter cultures in fresh LB medium, cultures were grown at 37°C to an OD600 of 0.8. Protein production was subsequently induced by adding  .1 mM IPTG and incubating at 16°C for 16 h with vigorous shaking. Following induction, the recombinant proteins were isolated from the harvested bacterial cells that had been resuspended in EBC buffer before sonication. The soluble fraction was obtained by removing insoluble material and cellular debris, then subjected to glutathione‐affinity purification through incubation with 50 µL of 50% glutathione‐Sepharose (Pierce) at 4°C for 3 h. Washed beads (3× PBS) yielded proteins that were either: (a) stored in PBS/10% glycerol at 4°C, or (b) eluted. Purity was confirmed by Coomassie staining, with BSA‐based quantification.

### 5.20 Western blot

The RIPA buffer was added to the cells or processed tissues at 4°C. Protein samples were loaded for electrophoresis, after that, carefully remove the gel and excise the target band(s) guided by the protein marker. Immerse the PVDF membrane in methanol for 1 min, then transfer to the transfer buffer. Then, blocking the PVDF membrane with 5% milk (1 h, room temperature) followed by primary antibody incubating overnight (4°C). The next day, wash the membrane by TBST, 15 min, three times. Then incubated with secondary antibodies for 1 h. Place the PVDF membrane on the imager, evenly apply the ECL working solution, remove air bubbles and initiate exposure. Each experiment was repeated in triplicate.

### 5.21 Antibodies

The custom PVT1‐104aa antibody was generated by first preparing the peptide immunogen through GenScript Biotech Corporation (Nanjing, Jiangsu, China), conjugated with KLH carrier protein and quality‐controlled via HPLC and MS to ensure >85% purity. Subsequently, two rabbits were immunized using GenScript's proprietary Polyexpress protocol over approximately 4 weeks; serum titre was tested after the third immunization to confirm a strong immune response before purification. Finally, antiserum was collected from each rabbit, pooled and affinity‐purified using the target peptide, with quality validation by indirect ELISA (1:500 for IB).

Antibodies against c‐Myc (#ab32072, 1:1000), MYC ser62 (#ab185656, 1:2000), MYC thr58 (#ab185655, 1:1000), GSK3β (#ab32391, 1:5000), FBXW7 (#ab192328, 1:1000) and myc tag (#ab32,1:1000) were from Abcam (Cambridge, MA, USA). Antibodies against β‐tubulin (#2128, 1:1000), β‐actin (#4967, 1:1000), GAPDH (#2118, 1:1000), Flag (#14793, 1:1000), HA (#3724, 1:1000), ubiquitin (#20326, 1:1000) and PD‐L1 (#13684, 1:1000) were from Cell Signaling Technology (Danvers, MA, USA).

### 5.22 Co‐immunoprecipitation

Harvest cells from 10 cm dishes, wash with ice‐cold PBS buffer, add IP lysis buffer supplemented with protease inhibitors, then lyse on ice for 30 min. Lysates were centrifuged, and the resulting supernatant was aliquoted for subsequent experiments. Co‐immunoprecipitation was performed using the Pierce Kit (Thermo), where beads were conjugated with the primary antibody to capture protein complexes. Wash the beads repeatedly with binding buffer to remove non‐specifically bound proteins. Bead‐bound complexes were denatured by heating at 95°C for 5 min in SDS‐PAGE loading buffer, and subsequently resolved by SDS‐PAGE for analysis.

### 5.23 Animal studies

Experimental rodents comprising C57BL/6J and BALB/c strains (6–8‐week‐old, sex‐balanced; GemPharmatech) were housed under standardized conditions maintaining 22–25°C ambient temperature with 12 h/12 h light‐dark photoperiods. Animal assignment to different study groups followed by computerized randomization protocols. Xenografts were established by subcutaneous dorsal injection of 5 × 10^5^ control or circPVT1‐knockdown MC38/CT26 cells in 100 µL ice‐cold PBS. Tumour size measurements and endpoint assessments were conducted by investigators blinded to the group assignments. Tumour volume was monitored via calliper measurements (*L* × *W* ^2^/2), with euthanasia performed upon reaching 15 mm diameter, moribund status, or exceeding permitted tumour burden. Lung metastasis models were generated by tail vein injection of MC38 cells (1 × 10^6^ cells suspended in 100 µL PBS), with formalin‐fixed lung tissues collected after 8 weeks for metastatic nodule enumeration. All quantifications were performed in a blinded manner. Treatment cohorts received twice‐weekly intraperitoneal injections of anti‐PD‐L1 (100 µg/dose; BioXcell #BP0101) or IgG control (#BE0083).

### 5.24 Immunohistochemistry

All tissue sections (xenografts and clinical specimens) underwent standard antigen retrieval with alkaline EDTA (pH 8.0) after deparaffinization. Peroxidase blocking was achieved with 3% H_2_O_2_ treatment. Samples were blocked using a solution of PBS with 5% normal goat serum (Vector) and 0.1% Triton X‐100 for 1 h following PBS rinses. Primary antibodies targeting c‐Myc (1:200), PVT1‐104aa (1:100), PD‐L1 (1:500) and Ki67 (1:2000) were applied at certain dilutions overnight. The HRP‐DAB detection system was employed for antigen localization.

### 5.25 Flow cytometry

Following euthanasia, mouse tumours were immediately harvested, minced into small fragments and enzymatically digested in DMEM containing Collagenase IV (1.5 mg/mL, Sigma) and DNase I (100 µg/mL, Sigma) at 37°C for 45 min with gentle agitation (250 rpm). Following digestion, the tissue was filtered through an 80 µm mesh to yield single cells. These cells then underwent centrifugation (300×*g*, 5 min), PBS washing and final resuspension in staining buffer containing 1% BSA and 1 mM EDTA in PBS. For immunophenotyping of immune cell subsets and exhaustion markers, single‐cell suspensions were labelled with a panel of fluorescently tagged monoclonal antibodies directed against specific surface antigens, including CD11b‐APC (MABF362, Sigma‐Aldrich), Gr‐1‐PE (MABF472, Sigma‐Aldrich), CD45‐PerCP‐Cy5.5(MABF160A, Sigma‐Aldrich), CD8‐PE (SAB4700088, Sigma‐Aldrich), CD4‐PE (MABF573, Sigma‐Aldrich), PD‐1‐APC (329907, Biolegend), Tim‐3‐FITC (11‐5870‐82, ThermoFisher), GZMB‐PE (12‐8988‐82, ThermoFisher) and PRF1‐PE (12‐9392‐82, ThermoFisher) according to the manufacturer's recommendations. For each staining panel, cells were incubated with antibodies for 30 min on ice in the dark. Samples were acquired on a BD FACSAria or equivalent flow cytometer after washing with staining buffer. Analysis was performed with FlowJo software. MDSCs were defined as CD45^+^CD11b^+^Gr‐1^+^ cells. The gating strategy defined CD4^+^ and CD8^+^ T cell populations within CD45^+^ lymphocytes, with their frequencies expressed relative to total viable cells. Surface and intracellular marker expression (PD‐1, Tim‐3, GZMB, PRF1) on CD8^+^ T lymphocytes was evaluated and quantified as MFI.

### 5.26 Chromatin immunoprecipitation PCR assay

Following the commercial protocol, we performed chromatin immunoprecipitation with the SimpleCHIP Enzymatic Chromatin IP Kit (Cell Signaling Technology). Following formaldehyde‐mediated cross‐linking and cellular lysis, chromatin was fragmented through limited micrococcal nuclease treatment. Chromatin immunoprecipitation was conducted using magnetic beads conjugated with c‐Myc‐specific antibodies or isotype control IgG. Following reversion of formaldehyde‐induced crosslinks, immunoprecipitated DNA underwent purification through silica‐membrane centrifugation and quantitative PCR assessment focusing on CD274 (PD‐L1) promoter sequences.

### 5.27 Statistical analysis

Data processing utilized GraphPad Prism 8.0 and Microsoft Excel 16.74, with results expressed as mean values ± standard deviation derived from triplicate experiments. Parametric comparisons employed: (1) paired *t*‐tests for tumour/normal tissue pairs; (2) unpaired *t*‐tests or ANOVA for other groups. Survival analysis used the Kaplan–Meier methodology. Significance thresholds were established at *p* < .05, with asterisk‐based annotation as follows: *p < .05, **p < .01, ***p < .001.

## AUTHOR CONTRIBUTIONS

Yiwei Li and Qingguo Li conceived the study concept, designed experimental approaches and supervised all research activities. Maoguang Ma, Mingdian Wanga and Yufei Yang collected the clinical samples and performed the experiments. Shaobo Mo and Sanjun Cai analyzed and interpreted the data. Maoguang Ma and Dakui Luo wrote the manuscript. Maoguang Ma, Yiwei Li and Weixing Dai conducted the statistical analysis.

## FUNDING INFORMATION

This work was supported by the National Natural Science Foundation of China (82203289 to M.M.) and the Youth Science Foundation of Zhongshan Hospital, Fudan University (2024ZSQN36).

## ETHICS STATEMENT AND CONSENT TO PARTICIPATE

The study was approved by the Ethics Institutional Review Boards of the Fudan University Shanghai Cancer Center and complied with all relevant ethical regulations regarding human participants (ethic no. 050432‐4‐2108*, 9 August 2021). All animal experiments were approved by the Animal Ethics Committee of Fudan University Shanghai Cancer Center (ethic no. FUSCC‐IACUC‐S2022‐0095, 21 February 2022). All experimental and animal procedures were followed by the guidelines and principles of the Helsinki Declaration and the International Council for Laboratory Animal Science (ICLAS).

## CONFLICT OF INTEREST STATEMENT

The authors declare no conflict of interest.

## Supporting information



Supporting Information

Supporting Information

Supporting Information

Supporting Information

Supporting Information

## Data Availability

The RNA‐seq data were deposited in the NCBI database under the accession ID: PRJNA1142914. The other data are available within the manuscript, Supporting Information Materials and Source data file. The data are available upon reasonable request.

## References

[1] Biller LH , Schrag D . Diagnosis and treatment of metastatic colorectal cancer: a review. JAMA. 2021;325:669‐685. 33591350 10.1001/jama.2021.0106

[2] Tan L , Peng D , Cheng Y . Significant position of C‐myc in colorectal cancer: a promising therapeutic target. Clin Transl Oncol. 2022;24:2295‐2304. 35972682 10.1007/s12094-022-02910-y

[3] Ye WL , Huang L , Yang XQ , et al. TRIM21 induces selective autophagic degradation of c‐Myc and sensitizes regorafenib therapy in colorectal cancer. Proc Natl Acad Sci USA. 2024;121:e2406936121. 39388269 10.1073/pnas.2406936121 PMC11494295

[4] Ghoussaini M , Song H , Koessler T , et al. Multiple loci with different cancer specificities within the 8q24 gene desert. J Natl Cancer Inst. 2008;100:962‐966. 18577746 10.1093/jnci/djn190 PMC2902819

[5] Sotelo J , Esposito D , Duhagon MA , et al. Long‐range enhancers on 8q24 regulate c‐Myc. Proc Natl Acad Sci USA. 2010;107:3001‐3005. 20133699 10.1073/pnas.0906067107 PMC2840341

[6] Guan Y , Kuo WL , Stilwell JL , et al. Amplification of PVT1 contributes to the pathophysiology of ovarian and breast cancer. Clin Cancer Res. 2007;13:5745‐5755. 17908964 10.1158/1078-0432.CCR-06-2882

[7] Ghesquières H , Larrabee BR , Casasnovas O , et al. A susceptibility locus for classical Hodgkin lymphoma at 8q24 near MYC/PVT1 predicts patient outcome in two independent cohorts. Br J Haematol. 2018;180:286‐290. 27716907 10.1111/bjh.14306 PMC5344766

[8] Riquelme E , Suraokar MB , Rodriguez J , et al. Frequent coamplification and cooperation between C‐MYC and PVT1 oncogenes promote malignant pleural mesothelioma. J Thorac Oncol. 2014;9:998‐1007. 24926545 10.1097/JTO.0000000000000202 PMC4287384

[9] Tseng YY , Moriarity BS , Gong W , et al. PVT1 dependence in cancer with MYC copy‐number increase. Nature. 2014;512:82‐86. 25043044 10.1038/nature13311 PMC4767149

[10] Fei Y , Cao D , Li Y , et al. Circ_0008315 promotes tumorigenesis and cisplatin resistance and acts as a nanotherapeutic target in gastric cancer. J Nanobiotechnol. 2024;22:519. 10.1186/s12951-024-02760-6 PMC11360491 39210348

[11] Li Y , Wang Z , Gao P , et al. CircRHBDD1 promotes immune escape via IGF2BP2/PD‐L1 signaling and acts as a nanotherapeutic target in gastric cancer. J Transl Med. 2024;22:704. 39080693 10.1186/s12967-024-05498-9 PMC11289934

[12] Hansen TB , Jensen TI , Clausen BH , et al. Natural RNA circles function as efficient microRNA sponges. Nature. 2013;495:384‐388. 23446346 10.1038/nature11993

[13] Pamudurti NR , Bartok O , Jens M , et al. Translation of CircRNAs. Mol Cell. 2017;66:9‐21.e7. 28344080 10.1016/j.molcel.2017.02.021 PMC5387669

[14] Zhong J , Yang X , Chen J , et al. Circular EZH2‐encoded EZH2‐92aa mediates immune evasion in glioblastoma via inhibition of surface NKG2D ligands. Nat Commun. 2022;13:4795. 35970825 10.1038/s41467-022-32311-2 PMC9378736

[15] Panda AC , Grammatikakis I , Kim KM , et al. Identification of senescence‐associated circular RNAs (SAC‐RNAs) reveals senescence suppressor CircPVT1. Nucleic Acids Res. 2017;45:4021‐4035. 27928058 10.1093/nar/gkw1201 PMC5397146

[16] Yi J , Wang L , Hu GS , et al. CircPVT1 promotes ER‐positive breast tumorigenesis and drug resistance by targeting ESR1 and MAVS. Embo J. 2023;42:e112408. 37009655 10.15252/embj.2022112408 PMC10183818

[17] Wang S , Su TT , Tong H , et al. CircPVT1 promotes gallbladder cancer growth by sponging miR‐339‐3p and regulates MCL‐1 expression. Cell Death Discov. 2021;7:191. 34312371 10.1038/s41420-021-00577-y PMC8313687

[18] Welcker M , Orian A , Jin J , et al. The Fbw7 tumor suppressor regulates glycogen synthase kinase 3 phosphorylation‐dependent c‐Myc protein degradation. Proc Natl Acad Sci USA. 2004;101:9085‐9090. 15150404 10.1073/pnas.0402770101 PMC428477

[19] Rottmann S , Wang Y , Nasoff M , Deveraux QL , Quon KC . A TRAIL receptor‐dependent synthetic lethal relationship between MYC activation and GSK3beta/FBW7 loss of function. Proc Natl Acad Sci USA. 2005;102:15195‐15200. 16210249 10.1073/pnas.0505114102 PMC1257707

[20] Casey SC , Tong L , Li Y , et al. MYC regulates the antitumor immune response through CD47 and PD‐L1. Science. 2016;352:227‐231. 26966191 10.1126/science.aac9935 PMC4940030

[21] Kim EY , Kim A , Kim SK , Y S . Chang MYC expression correlates with PD‐L1 expression in non‐small cell lung cancer. Lung Cancer. 2017;110:63‐67. 28676221 10.1016/j.lungcan.2017.06.006

[22] Le DT , Uram JN , Wang H , et al. PD‐1 blockade in tumors with mismatch‐repair deficiency. N Engl J Med. 2015;372:2509‐2520. 26028255 10.1056/NEJMoa1500596 PMC4481136

[23] Overman MJ , Lonardi S , Wong KYM , et al. Durable clinical benefit with nivolumab plus ipilimumab in DNA mismatch repair‐deficient/microsatellite instability‐high metastatic colorectal cancer. J Clin Oncol. 2018;36:773‐779. 29355075 10.1200/JCO.2017.76.9901

[24] Vo JN , Cieslik M , Zhang Y , et al. The landscape of circular RNA in cancer. Cell. 2019;176:869‐881.e13. 30735636 10.1016/j.cell.2018.12.021 PMC6601354

[25] Yang Y , Gao X , Zhang M , et al. Novel role of FBXW7 circular RNA in repressing glioma tumorigenesis. J Natl Cancer Inst. 2018;110:304‐315. 28903484 10.1093/jnci/djx166 PMC6019044

[26] Zhong J , Wu X , Gao Y , et al. Circular RNA encoded MET variant promotes glioblastoma tumorigenesis. Nat Commun. 2023;14:4467. 37491377 10.1038/s41467-023-40212-1 PMC10368723

[27] Li J , Ma M , Yang X , et al. Circular HER2 RNA positive triple negative breast cancer is sensitive to Pertuzumab. Mol Cancer. 2020;19:142. 32917240 10.1186/s12943-020-01259-6 PMC7488427

[28] Dhanasekaran R , Deutzmann A , Mahauad‐Fernandez WD , et al. The MYC oncogene—the grand orchestrator of cancer growth and immune evasion. Nat Rev Clin Oncol. 2022;19:23‐36. 34508258 10.1038/s41571-021-00549-2 PMC9083341

[29] D'Artista L , Moschopoulou AA , Barozzi I , et al. MYC determines lineage commitment in KRAS‐driven primary liver cancer development. J Hepatol. 2023;79:141‐149. 36906109 10.1016/j.jhep.2023.02.039 PMC10330789

[30] Zimmerli D , Brambillasca CS , Talens F , et al. MYC promotes immune‐suppression in triple‐negative breast cancer via inhibition of interferon signaling. Nat Commun. 2022;13:6579. 36323660 10.1038/s41467-022-34000-6 PMC9630413

[31] Klein IA , Resch W , Jankovic M , et al. Translocation‐capture sequencing reveals the extent and nature of chromosomal rearrangements in B lymphocytes. Cell. 2011;147:95‐106. 21962510 10.1016/j.cell.2011.07.048 PMC3190307

[32] Llombart V , Mansour MR . Therapeutic targeting of “undruggable” MYC. EBioMedicine. 2022;75:103756. 34942444 10.1016/j.ebiom.2021.103756 PMC8713111

[33] Yan S , Zhang G , Luo W , et al. PROTAC technology: from drug development to probe technology for target deconvolution. Eur J Med Chem. 2024;276:116725. 39083982 10.1016/j.ejmech.2024.116725

[34] Qin S , Xiao X . Key advances and application prospects of PROTAC technologies in the next 5 years. Future Med Chem. 2025;17:987‐989. 40314207 10.1080/17568919.2025.2498875 PMC12091913

[35] Han H , Jain AD , Truica MI , et al. Small‐molecule MYC inhibitors suppress tumor growth and enhance immunotherapy. Cancer Cell. 2019;36:483‐497.e15. 31679823 10.1016/j.ccell.2019.10.001 PMC6939458

[36] Yin Y , Liu B , Cao Y , et al. Colorectal cancer‐derived small extracellular vesicles promote tumor immune evasion by upregulating PD‐L1 expression in tumor‐associated macrophages. Adv Sci (Weinh). 2022;9:2102620. 35356153 10.1002/advs.202102620 PMC8948581

