# Supplementary material for: PVT1‐104aa derived from the 8q24 gene desert promotes colorectal cancer tumorigenesis
Source: Clin Transl Med. 2026 Apr 8;16(4):e70654. doi: 10.1002/ctm2.70654 (PMC13062635; doi:10.1002/ctm2.70654)
Supplement: Supplementary file 1 — Supporting Information [file CTM2-16-e70654-s003.docx]

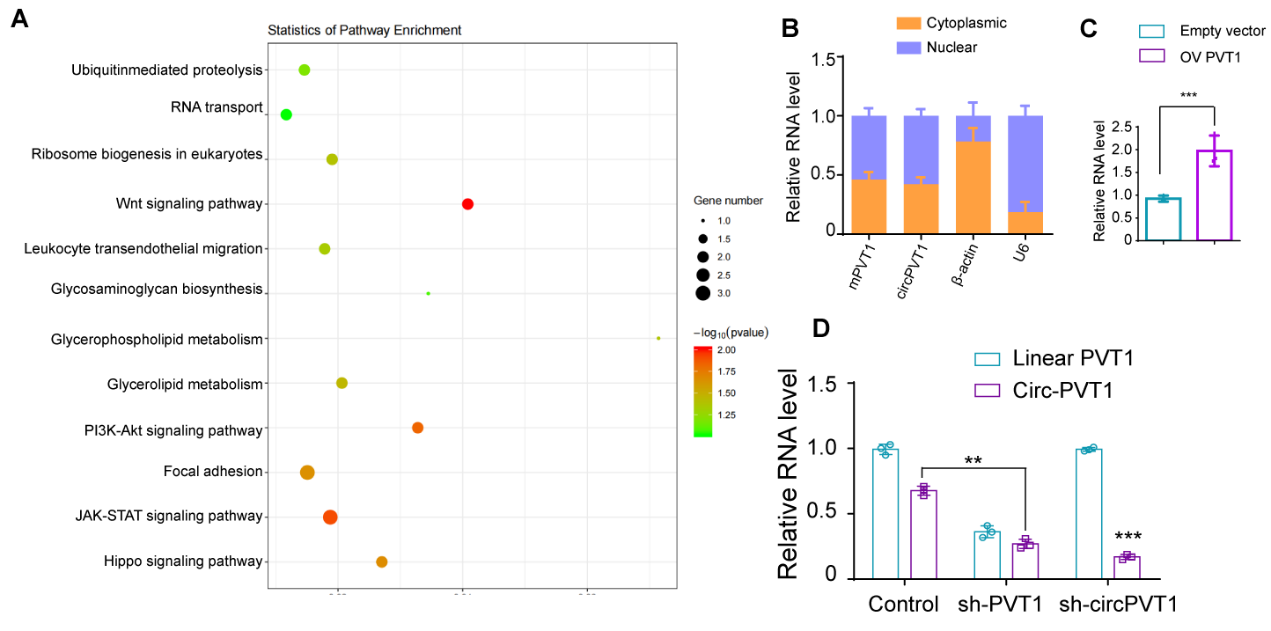


Supplementary figure 1

A. The comparative KEGG pathway enrichment analysis of target genes from upregulated miRNAs between groups is presented as a bubble plot. Each bubble represents an enriched pathway, with visual encoding of key analytical parameters: (1) Color gradient (green to red) indicates enrichment significance (p-value), where red denotes higher statistical significance; (2) Bubble size corresponds to the degree of gene overlap between the target genes and pathway genes; (3) The horizontal axis (Rich Factor) quantifies enrichment strength, calculated as the ratio of overlapping genes to the total number of genes in the pathway, with larger values indicating stronger pathway enrichment. B. The subcellular distribution of circ-PVT1 and linear PVT1 was assessed by analyzing RNA extracted from isolated cytoplasmic and nuclear fractions. β-actin and U6 RNA were used as markers for cytoplasmic and nuclear RNA, respectively. C. Following the overexpression of PVT1 mRNA in SW480 cells, the expression level of circPVT1 was analyzed by qPCR. D. Relative circ-PVT1 and linear PVT1 mRNA levels in lovo cells transfected with scramble shRNA or PVT1 shRNA or circ-PVT1 shRNA. The data are pooled from three independent experiments. The data are presented as the mean ±SD. Unpaired two-tailed Student’s t test was used to determine the significance of differences between the indicated groups where applicable. ***,p＜0.001; **, p＜0.01; *, p＜0.05.


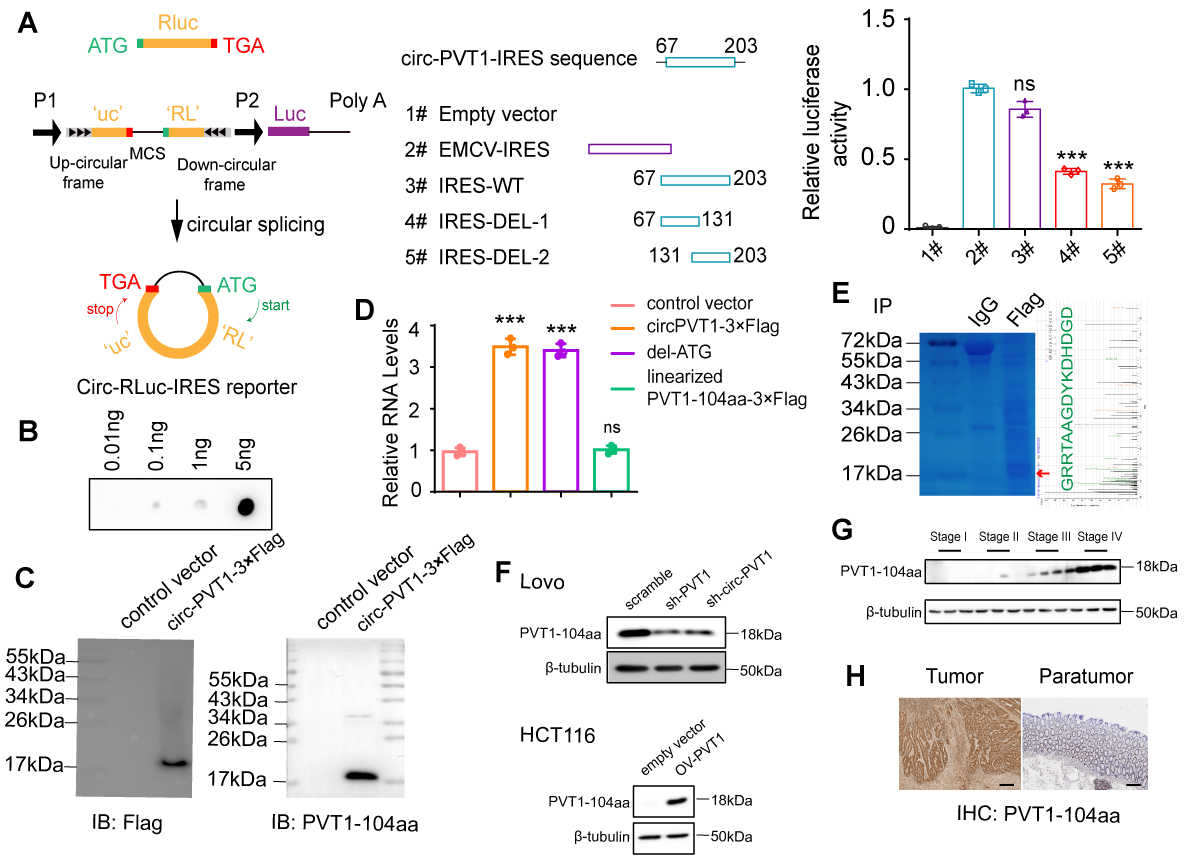


Supplementary figure 2

A. Identification and activity test of internal ribosomal entry site (IRES) in circ-PVT1 using circular vector-based luciferase reporter assay. EMCV-IRES was positive control. B. Immunoblot analysis of the specified synthetic peptides immobilized on a nitrocellulose membrane. C. Total proteins from circ-PVT1–3×Flag or control plasmid transfected HEK239T cells were prepared, and PVT1-104aa overexpression was confirmed by immunoblotting using Flag antibody or PVT1-104aa antibody. D. The relative expression levels of circPVT1 in cells overexpressing the above constructs. CircPVT1-3×Flag vs control, p＜0.0001; del ATG vs control, p=＜0.0001; linearized PVT1-104aa-3×Flag vs control, p=0.534. E. Total proteins from circ-PVT1 3×Flag or control plasmid-transfected HEK239T cells were separated via SDS-PAGE. The gel bands between 12 kD and 26 kD were cut and subjected to LC-MS/MS. The identified Flag-tag amino acids are shown in green. F, upper. lovo cells transfected with scramble shRNA or PVT1 shRNA or circ-PVT1 shRNA were subjected to immunoblotting with indicated antibodies; lower, overexpression of PVT1 mRNA in HCT116 cells led to a corresponding increase in PVT1-104aa protein levels. G. Expression levels of PVT1-104aa were detected in CRC tissues from stages I to IV. H. Representative IHC staining of PVT1-104aa in clinical tissue samples, scale bars, 250um. The data in (A) are pooled from three independent experiments. ***,p＜0.001; **, p＜0.01; *, p＜0.05.


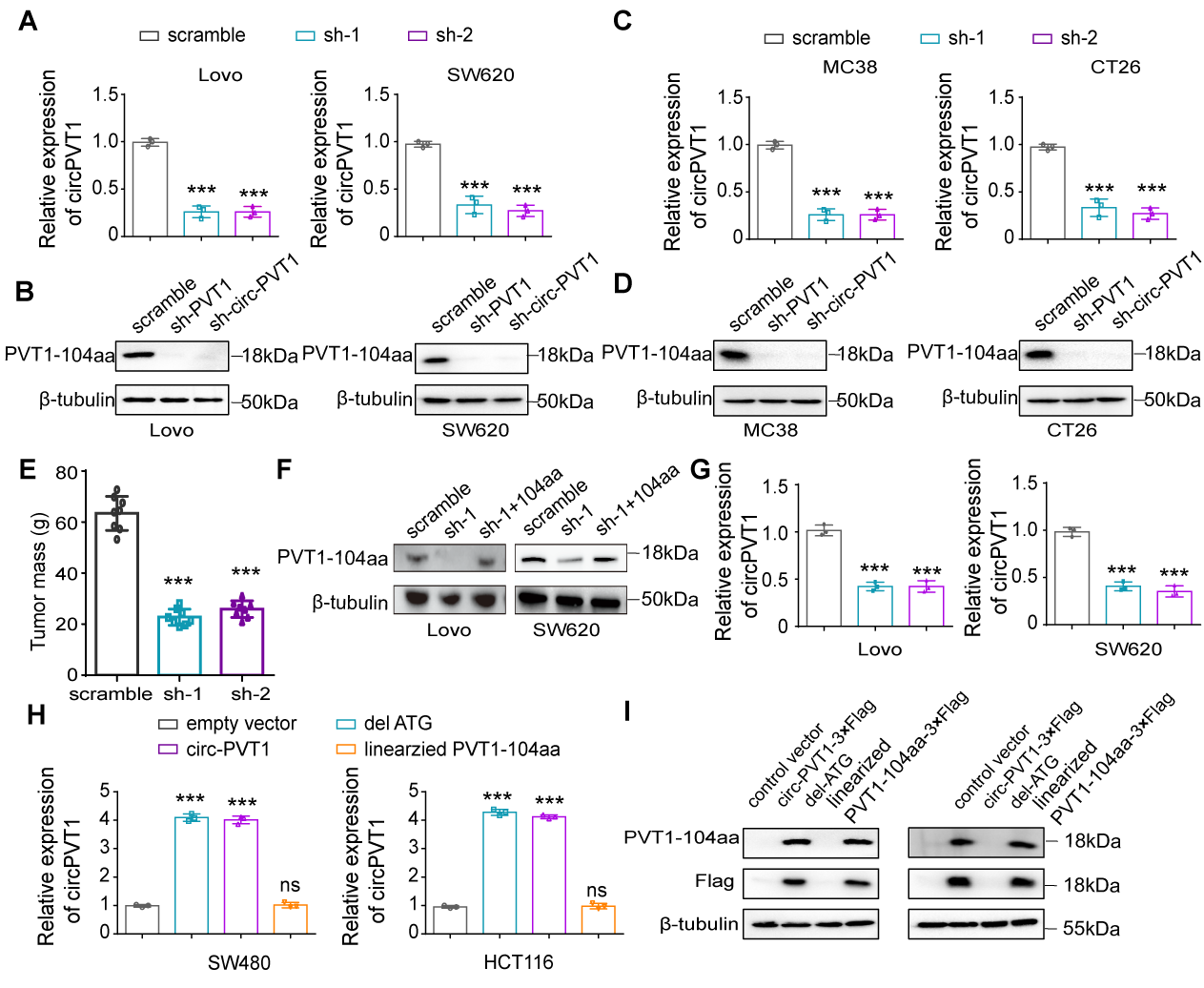


Supplementary figure 3

A, B. Lovo and sw620 cells were stably transfected with scramble or circ-PVT1 shRNAs, circ-PVT1 and PVT1–104aa expression levels were decided by q-PCR and immunoblotting. C, D. MC38 and CT26 cells were stably transfected with scramble or circ-PVT1 shRNAs, circ-PVT1 and PVT1–104aa expression levels were decided by q-PCR and immunoblotting. E. The weight of subcutaneous tumor was quantified as indicated. F, G. Lovo and sw620 cells were stably transfected with scramble or sh-1 or sh-1 with linearized PVT1-104aa plasmid, the circ-PVT1 and PVT1–104aa expression levels were decided by q-PCR and immunoblotting. H, I. Sw480 and HCT116 cells were stably transfected with empty vector, circ-PVT1*3Flag, del-ATG and linearized PVT1-104aa plasmid, the circ-PVT1 and PVT1–104aa expression levels were decided by q-PCR and immunoblotting. These data are pooled from three independent experiments. ***,p＜0.001; **, p＜0.01; *, p＜0.05.


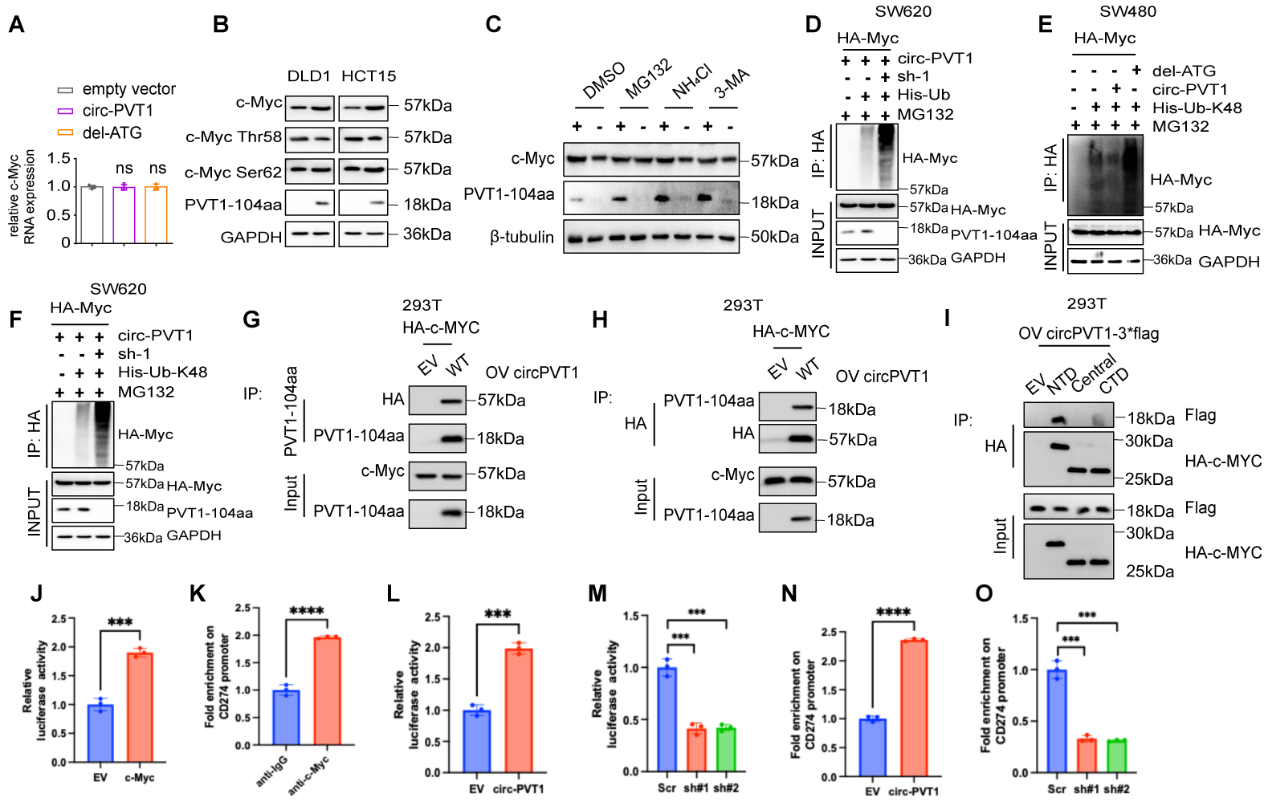


Supplementary figure 4

A. Relative c-Myc mRNA levels in sw480 cells transfected with empty vector, circPVT1 vector or del ATG vector. B. Following PVT1-104aa overexpression in DLD1 and HCT15 cells, c-Myc expression levels were analyzed by Western blot. C. HEK293T cells transfected with or without PVT1-104aa and treated with DMSO, MG123, NH4Cl or 3-MA were subjected to immunoblotting with indicated antibodies. D. WCL and Ub pull-down samples from SW620 cells (transfected as indicated and treated with 10 μmol/L MG132 for 12 h) were subjected to western blotting. E. Immunoprecipitation using HA antibody was performed in 293T cells with circ-PVT1 or del-ATG transfection after a 12-hour MG132(10 μM) treatment. F HA pull-down assays were conducted in Lovo cells expressing sh-circ-PVT1 or control constructs, following a 12-hour MG132 (10 μmol/L) treatment. G. Circ-PVT1 and HA-c-Myc was stably transfected to HEK293T cells, PVT-104aa was immunoprecipitated, followed by immunoblotting with antibodies against HA-c-Myc. H. circ-PVT1 and HA-c-Myc was stably transfected to HEK293T cells, HA-c-Myc was immunoprecipitated, followed by immunoblotting with antibodies against PVT1-140aa. I. HA-tagged c-Myc domains and flag-tagged PVT1-104aa were co-transfected into 293T cells. Coimmunoprecipitated truncated c-Myc protein (NTD) was detected by anti-HA antibody after immunoprecipitation with anti-Flag antibody. J. Luciferase activities of CD274 promoter reporter vectors in EV and overexpressed c-Myc Lovo cells. K. ChIP-qPCR assay showing the enrichment of c-Myc at CD274 promoter locus in Lovo cells. L. Luciferase activities of CD274 promoter reporter vectors in EV and overexpressed circ-PVT1 Lovo cells. M. ChIP-qPCR assay showing the enrichment of c-Myc at CD274 promoter locus in EV and overexpressed circ-PVT1 Lovo cells. N. Luciferase activities of CD274 promoter reporter vectors locus in Scr and sh-circ-PVT1 Lovo cells. O. ChIP-qPCR assay showing the enrichment of c-Myc at CD274 promoter locus in Scr and sh-circ-PVT1 Lovo cells. The data in (A-O) are pooled from three independent experiments. ***,p＜0.001; **, p＜0.01; *, p＜0.05.


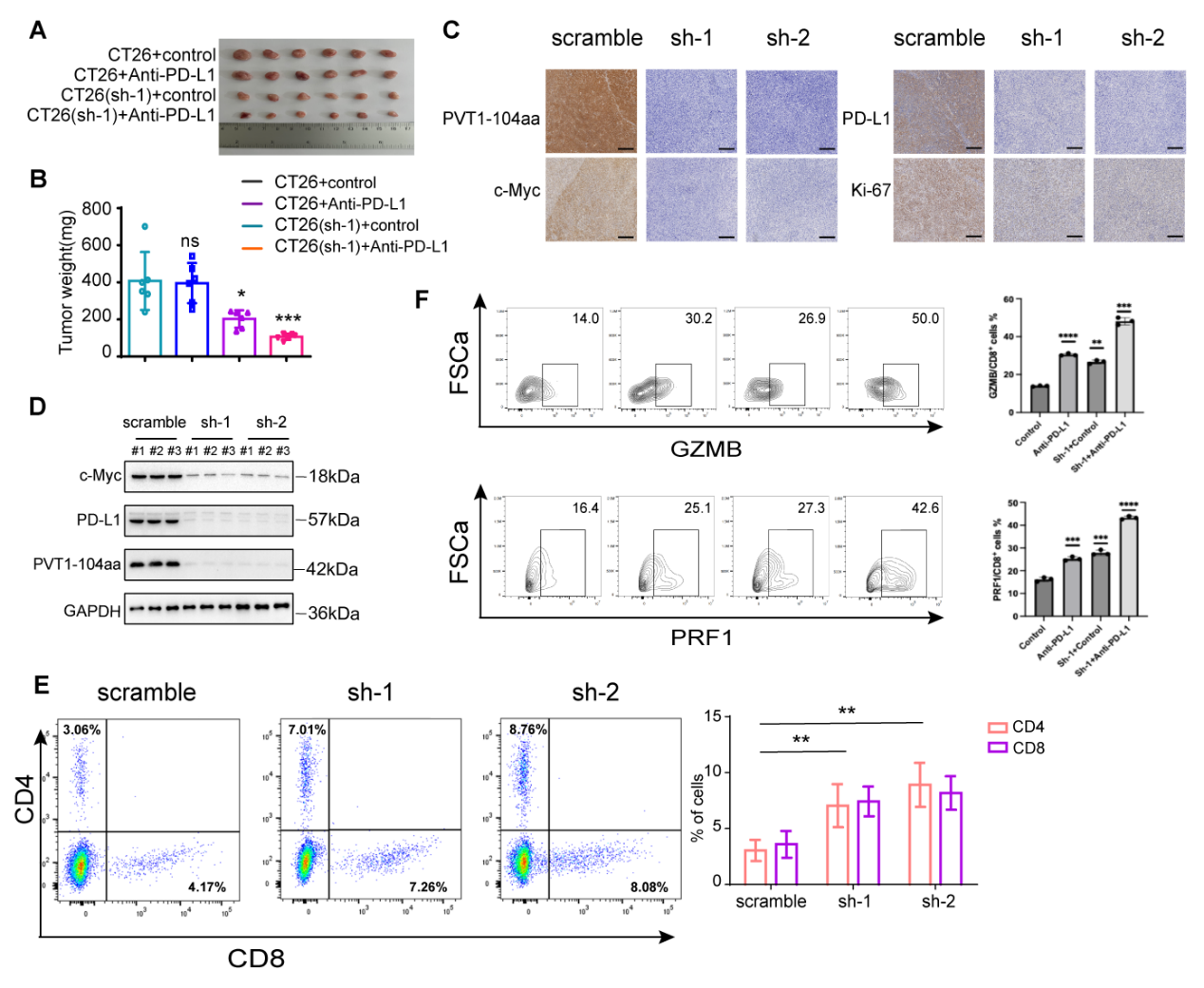


Supplementary figure 5

A. Representative image of dissected tumors from the CT26 syngeneic mouse model. B. Quantification of tumor weight across different experimental groups (mg). C. Representative IHC staining of PVT1-104aa, c-Myc, PD-L1 and Ki-67 in in each xenograft group as indicated. Scale bars, 50um. D. Tumors of each xenograft groups were subjected to immunoblotting with indicated antibodies. E. TILs were derived from the tumors of each xenograft groups, and the proportions of CD4+ andCD8+ T cells were measured by flow cytometry. F. The positive rates of GZMB and PRF1 among T cells in each group were detected using flow cytometry analysis. The data in (C-F) are pooled from three independent experiments. ***,p＜0.001; **, p＜0.01; *, p＜0.05.
